# Blood lipoprotein remodelling after non-severe paediatric burns: A longitudinal analysis beyond the acute phase

**DOI:** 10.1371/journal.pone.0362177

**Published:** 2026-11-12

**Authors:** Monique J. Ryan, Chloe Scott, Nathan G. Lawler, Reika Masuda, Jacqueline A. Davis, Nina D’Vaz, Lael M. Yonker, Maureen M. Leonard, Alessio Fasano, Luke Whiley, Jeremy K. Nicholson, Elaine Holmes, Fiona M. Wood, Samantha Lodge

**Affiliations:** 1 Centre for Computational and Systems Medicine, Health Futures Institute, Murdoch University, Perth, Western Australia, Australia; 2 School of Allied Health (Exercise Science), Murdoch University, Perth, Western Australia, Australia; 3 The Kids Research Institute, Perth Children's Hospital, Perth, Western Australia, Australia; 4 Mucosal Immunology and Biology Research Center, Department of Pediatrics, Massachusetts General Hospital, Boston, Massachusetts, United States of America; 5 Department of Paediatrics, Harvard Medical School, Boston, Massachusetts, United States of America; 6 School of Diagnostic and Therapeutic Sciences, Curtin University, Perth, Western Australia, Australia; 7 Curtin Medical Research Institute, Curtin University, Perth, Western Australia, Australia; 8 Dementia Centre of Excellence, enAble Institute, Curtin University, Perth, Western Australia, Australia; 9 Institute of Global Health Innovation, Faculty of Medicine, Imperial College London, London, United Kingdom; 10 School of Medicine, The Hong Kong University of Science and Technology, Hong Kong, China; 11 Section of Nutrition, Department of Metabolism, Digestion and Reproduction, Faculty of Medicine, Imperial College London, London, United Kingdom; 12 Burn Injury Research Unit, School of Biomedical Sciences, University of Western Australia, Perth, Western Australia, Australia; 13 Fiona Wood Foundation, Fiona Stanley Hospital, Murdoch, Western Australia, Australia; 14 WA Department of Health, Burns Service of Western Australia, Perth, Western Australia, Australia; 15 School of Medical, Molecular and Forensic Sciences, Murdoch University, Perth, Western Australia, Australia; 16 School of Biomedical Sciences, University of Western Australia, Perth, Western Australia, Australia; Institute of Medical Sciences, Banaras Hindu University, INDIA

## Abstract

Paediatric burn traumas are a global health concern shown to have long-term health consequences leading to increased morbidity and mortality. However, the underlying mechanisms contributing to these long-term health risks remain poorly understood. This study utilised ^1^H Nuclear Magnetic Resonance spectroscopy to comprehensively determine the lipoproteomic changes in the acute injury phase and long-term perturbations in blood samples longitudinally collected from 142 patients. In the acute phase of burn injury 55% of the lipoprotein main fractions and subfractions were significantly altered compared to non-burn controls. The plasma profiles from the burn injury group demonstrated increased LDL (low density lipoprotein) triglyceride concentrations (adjusted *p*-value = 2.12x10^-9^), an increased Apolipoprotein B100/Apolipoprotein A1 ratio (adjusted *p*-value = 4.29x10^-5^) and decreased HDL (High Density Lipoprotein, adjusted p-value = 1.16x10^-11^). At 1-year post-burn injury, it was found that 53% of the lipoprotein fraction and subfractions were altered compared to non-burn controls. The lipoproteomic profile showed a shift in LDL subfraction distribution, with a decrease in larger LDL subfractions (LDL subfraction 2 particle number, adjusted *p*-value = 2.52 × 10^−4^) and an increase in smaller LDL subfractions (LDL subfraction 6 particle number, adjusted *p*-value = 6.47 × 10^−5^), a pattern previously associated with cardiometabolic and cardiovascular risk. Significant lipid compositional changes of VLDL (very-low density lipoprotein), IDL (intermediate density lipoprotein), LDL and HDL were also observed at 1-year post-burn injury, notably the largest shifts were observed in IDL compared with non-burn controls. The proportion of cholesterol esters were markedly increased from 31.3% to 40.6%, free cholesterol increased from 12.8% to 15.1%, while phospholipids reduced from 29.5% to 23.3% and triglycerides reduced from 26.3% to 21.1%. Overall, this study demonstrates persistent lipoproteomic alterations following paediatric burn injury. These findings identify persistent metabolic perturbations that are not captured by conventional lipid measures, but further longitudinal studies incorporating inflammatory biomarkers and clinical cardiovascular outcomes are required to determine their clinical significance and relationship to future cardiovascular health.

## Introduction

Childhood burn traumas are a global health concern that lead to significant short- and long-term morbidities. In Australia, children under the age of 16 account for 30% of all burn-related hospital admissions, with similar statistics for other countries including the United States [1,2]. Although non-severe burns (affecting less than 20% of total body surface area (TBSA)) account for most paediatric burn admissions. Studies have shown that children may experience long-term physical and psychosocial complications years after the injury, even in the absence of scarring [3]. Burn injuries subject children to an increased risk of developing various health conditions, including stress disorders [4], gastrointestinal diseases [5], immune dysfunction [6] and cardiovascular disease [7]. However, the underlying mechanisms and pathology that predispose children with burn injuries to these long-term health risks remain poorly understood.

Emerging research suggests that long-term metabolic changes following paediatric burn trauma may be triggered by the hypermetabolic response during the acute phase of injury [8–10]. A common finding across these studies is altered lipid metabolism. Lipids, and the lipoproteins that act as their transport vehicle, are crucial to numerous biological functions. For example, the transportation of molecules [11] cell signalling [12] and inflammatory responses [13]. The size and composition of lipoproteins determine their physiological role. Increasing evidence suggests that lipoprotein class and structure are altered in the early stages of atherosclerosis and can influence the disease progression [14]. It is known that smaller, denser low-density lipoprotein (LDL) subfractions remain in the circulation longer because of their reduced affinity for LDL receptors. In addition, because they are small they can cross into the artery wall easily where glycation or oxidation increase their atherogenicity [15]. Recently, differences in circulating lipoproteins post-burn injury compared to non-burn controls have been reported in adults [16,17]. However, to date lipoprotein size and composition have not been comprehensively evaluated in children in the acute and post-injury phases, which could provide valuable insight into the pathophysiology underlying the long-term health risks associated with burn trauma.

Nuclear magnetic resonance (NMR) spectroscopy is one of the leading platforms for in-depth analysis of blood lipoproteins and their subfractions [18]. ^1^H NMR spectroscopy has been widely adopted to profile blood plasma in a plethora of diseases, such as cardiovascular disease [19], diabetes [20] and acute inflammatory conditions including sepsis [21] and SARS-CoV-2 infection [22]. Compared with conventional clinical measurements of total cholesterol, LDL, and HDL, this technology provides a more detailed lipoprotein profiling approach. It enables subclassification into HDL, intermediate-density lipoprotein (IDL), LDL, and VLDL fractions, further stratified within each class by density. This allows for a more nuanced assessment of subfraction composition, including apolipoproteins, free cholesterol, triglycerides, esterified and total cholesterol, and phospholipids within each lipoprotein class [18].

In this study we aim to conduct longitudinal sampling of paediatric burn patients to determine the lipoproteomic changes in the acute injury phase as well as longer term (>1-year post-burn) using ¹H NMR spectroscopy.

## Materials and methods

### Participant enrolment and sample collection

Participants were included from three distinct cohort studies:

#### Burns cohort.

Children (n = 142) were recruited from Perth Children’s Hospital (Perth, Western Australia) when undergoing treatment for an acute burn injury as a part of the Children’s Burn Injury Biobank. The participants underwent blood collections from February 2000 to Februaruy 2023 from children who were aged between 2 months and 15 years at the time of burn injury. Additional plasma samples were collected for some participants post-injury, resulting in a total of 197 samples. Informed written consent was provided by the participant's parent/guardian. The Child and Adolescent Health Service Ethics Committee (RGS0000001403) granted ethics permission for this study. For this study, we define acute burn injury as up to 14 days post-injury, the healing phase as 15 days to 1-year post-injury, and the recovered phase as beyond 1-year post-injury.

#### Australian control cohort.

Non-burn control children’s samples (n = 55) were obtained from the ORIGINS Project [23], which follows 10,000 children from womb to childhood [24]. Cryopreserved plasma samples were chosen for this study from children between one and three years of age. Children were enrolled at birth with written consent given from the parents and blood collections were completed March 2018 to February 2022. Ethics was approved via the Ramsay Health Care Western Australia / South Australia Human Research Ethics Committee (Reference: 2146W).

#### US control cohort.

Paediatric serum samples (n = 48) were obtained from the Massachusetts General Hospital Pediatric Biorepositories (Boston, MA, Principal Investigators: Dr. Maureen Leonard; MassGeneral Brigham IRB, 2016P000949 and Dr. Yonker MassGeneral Brigham IRB, 2020P000955). Informed written consent was provided by the participant's parent/guardian, and participant assent when age appropriate and blood collections were completed between February 2017 to November 2021. Ages ranged from 5 to 15 years. All procedures were completed in alignment with IRB approved protocols.

An overview of the cohort demographics can be found in the S1 Table in S1 File. The ethics for analysing all samples at the Australian National Phenome Centre within this study was approved by Murdoch University (Ethics No. 2021/160).

### ^1^H NMR sample preparation

All sample preparation and processing followed the guidelines recommended by Loo et al [25]. Serum/plasma samples were defrosted at room temperature for 1 hour prior to preparation for NMR analysis. Each sample was prepared as a mixture of phosphate buffer (75 mM Na_2_HPO_4_, 2 mM NaN_3_, 4.6 mM sodium trimethylsilyl propionate-[2,2,3,3-^2^H_4_] (TSP) in H_2_O/D_2_O 4:1, pH 7.4 ± 0.1) and serum/plasma at a 1:1 ratio for a final volume of 180 μL into the 3 mm SampleJet^TM^ NMR tubes and sealed with POM balls. Samples were manually shaken and stored at 5°C inside the SampleJet^TM^ automatic sample changer until measurement (< 24 h).

### ^1^H NMR spectroscopy data acquisition and processing parameters

NMR spectroscopic analysis of samples was performed on a 600 MHz Bruker Avance III HD spectrometer, equipped with a 5 mm BBI probe and fitted with the Bruker SampleJet^TM^ (Bruker BioSpin, Billerica, Massachusetts, USA) robot cooling system set to 5°C. A full quantitative calibration was completed prior to analysis using a protocol described elsewhere [26]. For each sample, a single one-dimensional (1D) experiment with solvent presaturation (128 scans, 98K data points, spectral width of 30 ppm) was completed with a total experimental time of 14 minutes and 53 seconds. Long term reference samples were analysed alongside study samples to ensure no batch effects. The 1D experiment was performed to quantify the blood lipoproteins using the Bruker *in vitro* Diagnostic Research (IVDr) Lipoprotein Subclass Analysis (B.I.LISA^TM^) method [18]. The B.I.LISA^TM^ method normalizes the spectra using Bruker’s QuantRef manager within Topspin and calibrates the spectra to the alanine doublet at 1.48 ppm. The -CH_3_ (δ 0.80) and -CH_2_- (δ 1.25) groups are then fitted using a PLS-2 regression model. The resulting 112 parameters consist of total plasma lipid analytes, cholesterol, free cholesterol, phospholipids, triglycerides, apolipoproteins A1/A2/B100 and the distributions within different density classes of plasma-lipoproteins. High-density lipoprotein (HDL, 1.063–1.210 kg/L), low-density lipoprotein (LDL, 1.019–1.063 kg/L), intermediate-density lipoprotein (IDL, 1.006–1.019 kg/L) and very low-density lipoprotein (VLDL, 0.950–1.006 kg/L). The main lipoprotein classes HDL, LDL and VLDL are subdivided further by density class. LDL subdivisions: LDL1 (1.019–1.031 kg/L), LDL2 (1.031–1.034 kg/L), LDL3 (1.034–1.037 kg/L), LDL4 (1.037–1.040 kg/L), LDL5 (1.040–1.044 kg/L) and LDL6 (1.044–1.063 kg/L). HDL subdivisions: HDL1 (1.063–1.100 kg/L), HDL2 (1.100–1.112 kg/L), HDL3 (1.112–1.125 kg/L) and HDL4 (1.125–1.210 kg/L) and the VLDL subfractions were subdivided into 5 density classes. A full list of the subfractions and parameter annotations can be found in the S2 Table in S1 File.

### Data Analysis

All computation and data visualisation were performed using R (version 4.4.3) and Rstudio IDE (version 3.6.0) (Team, R.C. (2024). R: A Language and Environment for Statistical Computing). Multivariate analyses and associated visualisations were created via *mva.plots* (“phenological/mva-plots version 0.0.8") [27]. Pie charts demonstrating changes in lipoprotein lipid composition were visualised using *uva.plots* (“phenological/uva-plots version 0.0.1") [28]. Group comparisons were performed using orthogonal projection to latent structures-discriminant analysis (OPLS-DA). Models were constructed using one predictive component, while the number of orthogonal components was determined automatically by the algorithm.

Model performance was evaluated using the cumulative proportion of variance explained in the response variable (R^2^Y) and the predictive ability estimated by seven-fold cross-validation (Q^2^). To assess model robustness and the potential for overfitting, permutation testing was performed using 1000 random permutations of the class labels. For each permutation, an OPLS-DA model was refitted and compared with the original model. Statistical significance was determined using empirical permutation *p*-values for both R^2^Y and Q^2^. The original model was considered robust when its performance significantly exceeded that of the permuted models. A permutation plot was generated to visualize the distribution of R^2^Y and Q^2^values obtained from the permuted models relative to the original model. The Cliff’s delta was calculated for all OPLS-DA models to determine the overall effect size for the intergroup differences visualised within an eruption plot [29]. For all multivariate analyses, non-burn controls were matched to burn participants using a 1:1 exact matching strategy based on age and sex. Matching was performed manually, with each burn participant paired with a non-burn control of the same sex and the same chronological age (in years). As exact matching was used, no age calliper or allowable age range was applied. The number of participants included in each matched analysis is provided in the corresponding figure legends. This matching strategy ensured that there were no significant differences in mean age or sex distribution between the burn and non-burn groups. The Wilcoxon signed-rank test (Mann-Whitney-U) was performed for univariate comparisons, and *p*-values were adjusted for multiple testing using the Benjamini and Hochberg method.

### Lipoprotein lipid composition

The total lipid composition of VLDL, IDL, LDL and HDL were determined by summing the concentrations of triglycerides (TG), cholesterol (CH) and phospholipids (PL). The cholesterol ester (CE) concentration for each fraction was derived indirectly by subtracting free cholesterol (FC) from total cholesterol (CE = CH − FC), under the assumption that total cholesterol reflects the combined pool of esterified and unesterified cholesterol measured within the same analytical platform. This method relies on the accurate and independent quantification of both total and free cholesterol by the assay and assumes negligible contribution from other cholesterol-related species or analytical interference. We acknowledge that this indirect calculation may propagate analytical variability from both measurements and does not account for potential heterogeneity within esterified cholesterol subfractions. This provides an estimated measure of the esterified cholesterol fraction within each lipoprotein class. All TG, CH, PL and FC concentrations were provided by the B.I.LISA^TM^ method.

## Results and discussion

*Cohort demographics:* To assess potential heterogeneity between geographical collection sites in the non-burn control cohorts (USA and Australia), lipoproteomic profiles were adjusted for age prior to multivariate analysis. Unsupervised principal component analysis (PCA) demonstrated no clear clustering of samples according to geographical origin (S1 Fig in S1 File), suggesting that site-specific differences were not a dominant source of overall lipidomic variation. Furthermore, OPLS-DA was performed to determine whether geographical origin could be predicted from the lipidomic profiles. No significant predictive model was generated, with the first predictive component failing to reach significance. The resulting OPLS-DA model demonstrated poor predictive performance (R^2^Y(cum) = 0.063; Q^2^(cum) = −0.059), and permutation testing confirmed that the model was not significant (pR^2^Y = 0.95; pQ^2^ = 0.15). Together, these findings indicate that geographical origin did not generate a robust or discriminative lipoproteomic signature within the control cohort.

Samples in the paediatric burn cohort had a median age of 3.59 years, ranging from 6 months to 15 years at the time of non-severe burn injury, with boys comprising 56.34% of the cohort. The median TBSA was 2%, where scald was the most common burn type (46.48%) and 86.62% of participants required burn debridement surgery. With the maximum TBSA at 7.85%, the burn injury cohort was classified as non-severe. Full cohort demographics can be found in S1 Table in S1 File.

*Acute burn injury in children:* OPLS-DA modelling comparing acute burn injury and the non-burn group revealed a discriminant lipoprotein signature where 55% of the lipoprotein main fractions and subfractions were found to be significantly different after multiple testing (Fig 1, S3 Table in S1 File). The plasma profiles from the burn injury group demonstrated higher LDL TG concentrations (LDTG, adjusted *p*-value = 2.12x10^-9^), with most of the LDL TG subfractions weighting highly in differentiating the burn group (L1TG, adjusted *p*-value = 2.12x10^-13^ to L6TG, adjusted *p*-value = 2.00x10^-10^). High concentrations of LDL TGs have been shown to be significantly increased in adults in inflammatory conditions associated with acute infection such as COVID-19 [30] and hepatitis C [31]. The Apolipoprotein B100/Apolipoprotein A1 ratio (ABA1), a lipoprotein-based biomarker that has been associated with atherosclerotic risk [32,33] was markedly higher in the acute phase of burn injury (adjusted *p*-value = 4.29x10^-5^). Elevated ABA1 has also been reported in children with obesity [34] and in children with acute COVID and Multisystem Inflammatory Syndrome in Children (MIS-C) [35].

**Fig 1:**
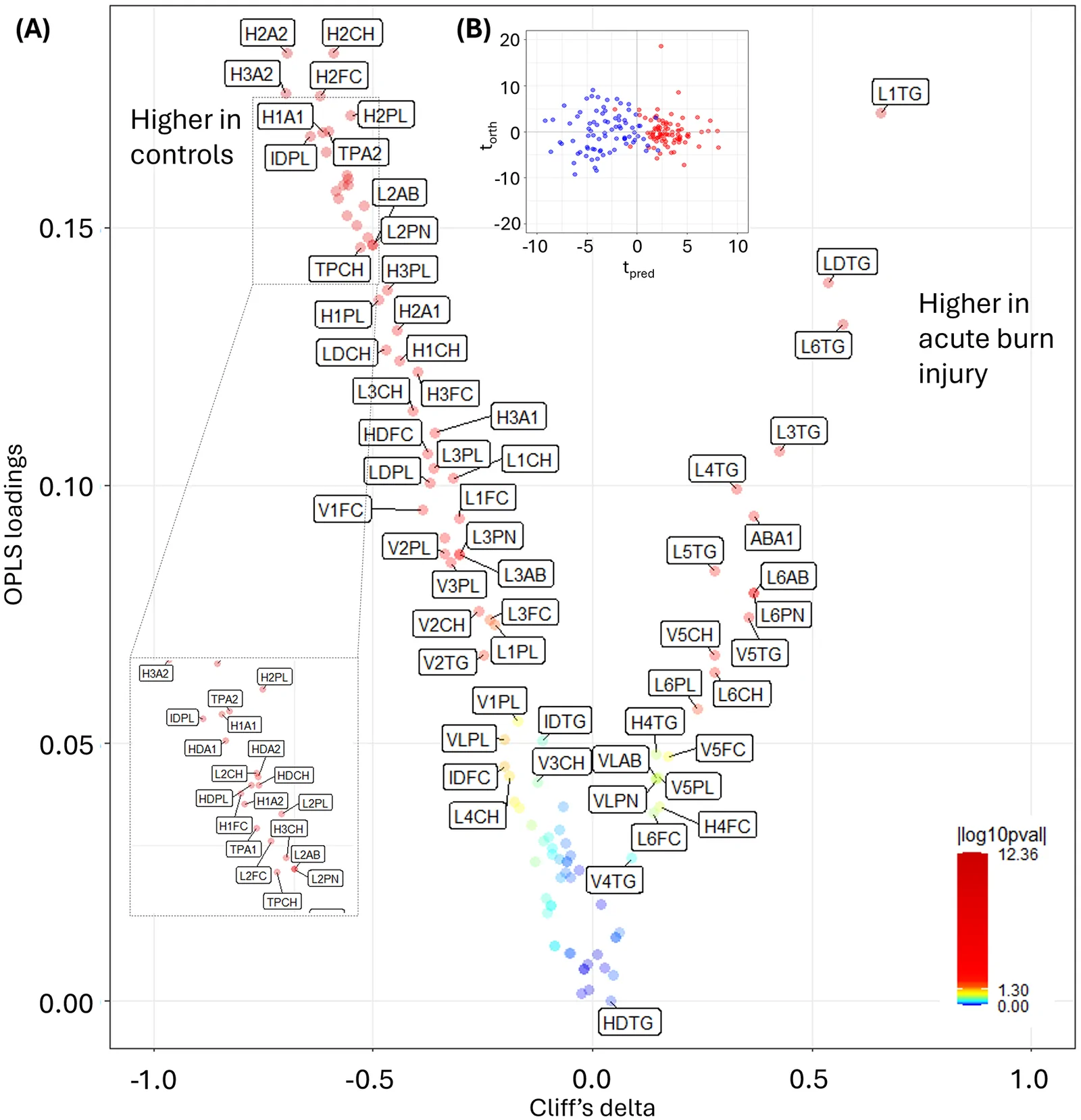
Eruption plot with OPLS-DA scores of acute burn injury (n = 86) and non-burn controls (n = 86) for the blood lipoproteins. **(A)** Eruption plot for acute burn injury and controls. **(B)** OPLS-DA scores plot for the control (blue) and acute burn injury (red) (R^2^X = 0.79, R^2^Y = 0.68, Q^2^Y = 0.62). Adjusted *p*-values, OPLS-DA loadings and Cliff’s delta values can be found for all lipoprotein parameters in S3 Table in S1 File. Validation of the model by permutation testing is shown in S2 Fig in S1 File.

Conversely, total HDL concentration was lower in the burn injury group, driven by lower concentrations of apolipoprotein A1 (apoA1) in HDL (HDA1; adjusted *p*-value = 1.16x10^-11^). Since there is one apoA1 molecule in each HDL particle, the apoA1 concentration can be used as a proxy for particle number. When stratified according to density, the plasma concentrations of apoA1 were depleted in the burn injury group. ApoA1 is the main structural protein of HDL. Apolipoprotein A2 (ApoA2) in HDL subfractions 1–3 (H1A2, adjusted *p*-value = 1.08x10^-10^; H2A2, adjusted *p*-value = 7.83x10^-15^; H3A2, adjusted *p*-value = 6.48x10^-15^) was significantly lower in comparison with non-burn controls (Fig 1). The role of apoA2 is more controversial. It has been shown to have an anti-inflammatory and antioxidant role and is therefore considered a protective biomarker [36]. ApoA2 has been found to be reduced in patients with other inflammatory conditions such as sepsis [21] and pulmonary arterial hypertension [37]. However, in mice overexpression of apoA2 has been shown to convert HDL from an anti to pro inflammatory factor, a change associated with reduced levels of paraoxonase within the HDL particles [38].

The ratio of the sum of HDL1 to HDL4 was highly significant between the burn versus control group (adjusted *p*-values = 1.80x10^-9^). This ratio has been shown previously to have an inverse association with BMI, which is significant in various inflammatory [39]. Lower IDL plasma PL concentrations (IDPL, adjusted *p*-value = 6.43x10^-13^) were one of the most significant features of burn injury. Notably, concentrations of the larger, less dense LDL subfractions 2 and 3 were also significantly lower in the burn injury group (e.g., for LDL subfraction 2 particle number (L2PN): Cliff’s delta = −0.5 (adjusted *p*-value = 2.45x10^-8^)). Larger LDL particles are less atherogenic as they are less likely to enter and accumulate in artery walls and therefore considered less harmful than the smaller LDL particles. Thus, reduction of these larger LDL subfractions in the acute burn injury phase is consistent with a lipoprotein profile that has previously been associated with greater atherogenic potential [40].

In summary, during the acute phase of the burn injury in children, we observed a dysregulated lipoprotein profile characterised by higher concentrations of LDL TGs and an increased ABA1 ratio, together with lower concentrations of HDL. It is known that severe physiological stress following tissue injury activates catecholamine, glucocorticoid and inflammatory signalling, increasing energy expenditure and promoting mobilisation of endogenous energy substrates. Increased adipose tissue lipolysis increases the delivery of free fatty acids to the liver, where they can contribute to hepatic triglyceride synthesis and incorporation into triglyceride rich lipoproteins. At the same time, inflammation and altered lipoprotein lipase activity can influence the production, remodelling and clearance of circulating lipoproteins [41,42]. The reduction in apoA1-containing HDL particles may similarly reflect inflammation associated remodelling of HDL metabolism during the acute stress response. HDL is dynamically remodelled through interactions with lipid transfer proteins, lipases and hepatic receptors, and inflammatory signalling can alter both HDL composition and its associated proteins. Consequently, reductions in apoA1 and changes in HDL subfractions may represent broader changes in lipid transport and HDL remodelling during burn induced metabolic stress [43]. Importantly, the present study measured lipoprotein concentrations and composition rather than HDL function; therefore, altered HDL quantity should not be interpreted as evidence of impaired HDL function or reduced cholesterol efflux capacity.

We recently investigated the lipidomic impact of non-severe burn injury in adults [16], in which the lipoprotein signature was characterised. Comparing this adult population with the children in the current study, clear differences in response to burn injury were noted. Of the eight most significantly reduced lipoproteins in the adult burn cohort, only four were significantly altered in the children: total plasma apolipoprotein A1 (TPA1), HDL apolipoprotein A1 (HDA1), HDL apolipoprotein A2 (HDA2) and total plasma apolipoprotein A2 (TPA2). The significantly lower concentrations of plasma HDL subfraction 4 CH (H4CH), LDL subfraction 4 FC (L4FC), LDL subfraction 4 particle number (L4PN) and HDL subfraction 4 apolipoprotein A1 (H4A1) seen in the adult’s post-burn injury was not replicated in the paediatric cohort.

Similarly, there was only a partial overlap in the subset of lipoproteins that were differentially higher following burn injury between the adult and child cohorts compared to non-burn controls. Of the top ten most significant lipoproteins found in higher concentrations in adults after burn injury, seven were significant in children: VLDL subfraction 5 PL (V5PL), VLDL subfraction 5 CH (V5CH), VLDL subfraction 5 TG (V5TG), LDL subfraction 2 particle number (L2PN), LDL subfraction 2 apolipoprotein B100 (L2AB), LDL subfraction 2 PL (L2PL) and ABA1. In contrast, VLDL subfraction 4 TG (V4TG), IDL particle number (IDPN) and LDL subfraction 2 TG were modulated in adults but not in children following burn injury. VLDL is not routinely measured clinically as it generally requires time consuming analyses such as gradient gel electrophoresis, ion mobility analysis or ultracentrifugation and TG concentrations serve as an estimate of VLDL levels [41]. Typically, high VLDL levels are associated with increased chronic disease risk in adults [19]. The ABA1 ratio is also known to increase in other acute inflammatory conditions such as COVID-19 infection [30] and in some chronic diseases including obesity, diabetes [20] and cardiovascular disease [19]. Here it is suggestive of a systemic impact after an acute burn injury in children consistent with observations in an adult cohort [16].

In children, LDL TGs were the most significantly increased across the subfractions. Within the LDL class, a shift towards a higher proportion of smaller LDL particles was also observed (LDL6 particle number was higher in acute burn injury compared to the non-burn controls). Both the shift towards higher density LDL particles and increased TG levels have been associated with inflammation. One potential mechanism is triglyceride enrichment of apoB-containing particles followed by lipolytic remodelling, which can promote changes in LDL particle size and density [44,45]. This shift towards smaller, denser LDL subfractions, observed in children but not adults, is consistent with lipoprotein alterations that have previously been described in inflammatory states. For example mild COVID-19 symptoms in children have the same lipoproteomic changes as in severe COVID-19 infection in adults [35].

The partial overlap between paediatric and adult burn lipoproteomic signatures may reflect differences in the magnitude and duration of the metabolic response, as well as age dependent lipid metabolism. Burn injury induces hypermetabolism, hormonal stress responses, lipolysis and altered hepatic lipid handling, which can affect triglyceride synthesis, VLDL secretion and lipoprotein remodelling. Inflammation may further modify lipoprotein metabolism through changes in lipoprotein lipase activity and HDL remodelling. Thus, differences between children and adults may reflect distinct metabolic adaptations rather than a uniform burn associated lipoprotein response. However, these mechanisms remain speculative, as relevant hormonal, inflammatory and lipoprotein kinetic measures were not assessed.

*Long-term lipoprotein perturbations post-acute burn injury:* To assess any long-term blood lipoprotein changes following burn injury, the non-burn controls were compared to the healing phase group (14 days > 1-year post-injury) (S4 Table in S1 File) and recovered phase (1-year post-injury) (S3-S25 Figs in S1 File). It was found that 53% of the lipoprotein main and subfractions were significantly different in the recovered group compared to the non-burn control group (Fig 2, S5 Table in S1 File).

**Fig 2:**
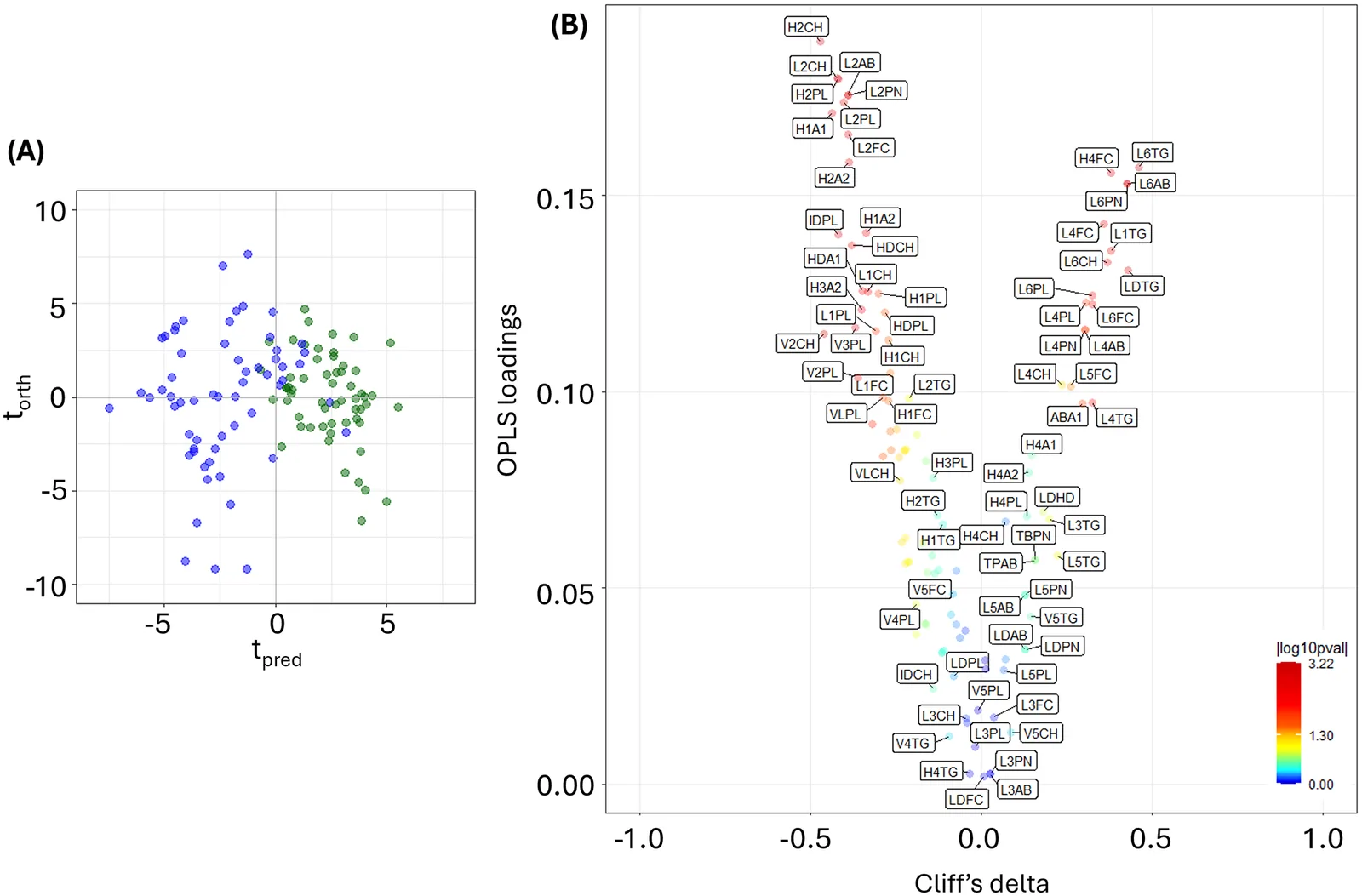
Eruption plot with OPLS-DA scores of the recovered burn injury group (n = 58) and matched age and sex non-burn samples (n = 58), and model loadings for the blood lipoproteins. **(A)** OPLS-DA scores plot for the control (blue) and ‘recovered’ burn injury (green) (R^2^X = 0.82, R^2^Y = 0.63, Q^2^Y = 0.51). The corresponding adjusted *p*-values, OPLS-DA loadings and Cliff’s delta values can be found for all lipoprotein parameters in Supplementary Table S5 in S1 File. **(B)** Eruption plot for matched non-burn controls (higher levels – left) and recovered participants (higher levels – right). Validation of the model by permutation testing is shown in S26 Fig in S1 File. Univariate comparison of the non-burn controls, acute phase, healing phase and recovered phase of the 112 measured lipoprotein parameters can be found in S3-S25 Figs in S1 File and the associated Supplementary Tables.

As in the acute phase of burn injury a shift in the particle numbers of the LDL subfractions was observed in the recovered group where the LDL subfraction 2 (L2PN) was reduced and LDL subfraction 6 (L6PN) was increased in the recovered burn injury participants compared to the non-burn injury controls. Additionally, LDL TG (LDTG, adjusted *p*-value = 5.66x10^-5^), LDL subfraction 6 particle number (L6PN, adjusted *p*-value = 6.47x10^-5^) and LDL subfraction 6 apolipoprotein B100 (L6AB, adjusted *p*-value = 6.47x10^-5^) were significantly increased compared to non-burn controls. These shifts were attributed to relatively higher particle numbers of small dense lipoproteins rather than an inherent compositional change in the components of the lipoprotein particles (S5 Fig in S1 File). In contrast, LDL subfraction 2 particle number (L2PN, adjusted *p*-value = 2.52x10^-4^) was significantly lower in the recovered group. Interestingly, there appeared to be some degree of normalisation of LDL subfraction 2 to the non-burn control concentrations (S5B Fig in S1 File) as demonstrated by the reduction of Cliff’s delta through the acute (cd = 0.50), healing (cd = 0.45) and recovered (cd = 0.39) phases in comparison to the non-burn controls. Due to a reduction in the particle number of LDL subfraction 2, a corresponding decrease in lipid concentrations was observed. This was reflected in the loadings plot of the model, which showed lower levels of cholesterol (L2CH), phospholipids (L2PL), apolipoprotein B100 (L2AB), and free cholesterol (L2FC) within this subfraction.

One of the most significant differences between the recovered phase in comparison to the non-burn controls was the significantly lower concentrations of HDL subfraction 2 CH (H2CH, cd = −0.47, adjusted *p*-value = 1.03x10^-5^). Although the reduction was significant, there was a gradual increase over time that appeared to be nearing non-burn control concentrations (S21B Fig in S1 File; non-burn controls mean concentration = 9.94 mg/dL, acute burn injury phase mean = 7.02 mg/dL, healing phase mean = 7.91 mg/dL, recovered phase mean = 8.44 mg/dL). Concentrations of HDL subfraction 1 were also significantly lower (H1A1, *p*-value = 4.19x10^-5^) in the recovered paediatric patients when compared to the non-burn controls and to the acute burn injury samples, which was confirmed by the reduced apolipoprotein A1 in HDL subfraction 1 in the recovered group. In conjunction, HDL subfraction 4 FC (H4FC) was found to be significantly higher 1-year post-burn injury (*p*-value = 3.77x10^-4^). Notably, H4FC was not significantly different at the acute burn injury phase from non-burn controls, (S22D Fig in S1 File) but increased in the healing phase to become significantly different compared to non-burn controls (adjusted *p*-value = 5.70x10^-5^). This phenomenon of new metabolic states emerging during ‘recovery’ that are neither present in acute disease conditions nor in the control state has been seen previously in COVID-19 infection in adults, where 3-indole acetic acid was not significantly altered in the acute phase of infection but significantly increased 3 months post recovery [46].

Further investigation into this phenomenon revealed another subset of lipoprotein parameters that were not significantly different from non-burn controls in the acute burn injury phase but became significantly different over time. For example, total VLDL particle number (VLPN) was not significantly different in the acute phase of burn injury but was significantly lower than in non-burn controls in the recovered phase (S4B Fig in S1 File, adjusted *p*-value = 7.50x10^-3^). VLDL subfractions 1–3 were lower in the acute burn group compared to controls and continued to decline during the healing and recovered phases (Fig 2, S4 and S5 Tables in S1 File). However, VLDL subfraction 4 was predominantly unchanged in the acute phase but began reducing in the healing phase and only became significantly reduced in the recovered phase. On the contrary, VLDL subfraction 5 remained at non-burn control levels during the healing and in the recovered phase. A reduction in VLDL CH has typically been associated with beneficial impact in reducing cardiovascular risk both in adults [47], and children with obesity [48], although another study found that a rearrangement of VLDL lipid content was prognostic for 1-year mortality in acute heart failure [49]. Reduced VLDL levels have also been observed in patients with metabolic syndrome due to the condition leading to liver fibrosis and inflammation. It was shown that increased insulin sensitivity and low liver synthetic function was associated with reduced VLDL production and circulating VLDL particle numbers [50]. The reduction in VLDL observed in the recovered cohort could reflect altered hepatic VLDL production, changes in hepatic lipid availability, or increased peripheral clearance and remodelling of VLDL particles. Burn-associated alterations in adipose tissue lipolysis, hepatic substrate utilisation and systemic energy expenditure provide plausible mechanisms through which VLDL metabolism could remain altered beyond the acute injury phase.

Cumulatively, these findings suggest distinct acute and longer-term phases of lipoprotein remodelling following burn injury. Several alterations observed during the acute phase partially normalised, whereas others persisted or emerged during the healing and recovered phases. The emergence of alterations that were not prominent during the acute phase may reflect continued metabolic adaptation following the initial injury, rather than a simple return to the pre-injury metabolic state. This phenomenon has been observed previously in adults with significant inflammatory conditions, for example in patients with respiratory distress syndrome that have developed new conditions and functional disability within five years of the initial event [51].

*Long-term alterations in lipoprotein particle number post-burn injury:* Quantitative analysis of circulating lipoproteins is critical to understanding the health status of individuals [52,53], particularly those that may be living with obesity and a predisposition to non-communicable diseases such as cardiovascular disease and type 2 diabetes [19,54]. The standard care model involves patients undergoing a lipid blood test, which measures total CH, HDL CH and total TG and uses the Friedwald equation to obtain LDL CH values [55]. When comparing the’recovered’ children to the matched non-burn controls, their total CH, HDL CH, and LDL CH were not significantly different, except for total TGs (adjusted *p*-value = 0.04).

When assessed at a typical health clinic, these 1-year post-burn injury children would appear to lie within normal ranges via clinical lipid measures and thus would not raise any concerns. However, we illustrate significant changes in the distribution of lipoprotein main and subfractions, which were persistently altered post-injury and is a finding that would not be detected using traditional clinical assessments. VLDL and IDL were found to be significantly reduced (S4 Fig in S1 File) while total HDL and LDL remained unchanged. However, it cannot be determined in this study if the reduction in VLDL may be due to reduced hepatic production or from increased catabolism of the lipoprotein particles [56]. Within the LDLs, LDL subfraction 2 was significantly reduced while subfraction 6 was significantly increased (Fig 3). This demonstrates a shift from larger, more buoyant LDL subfractions towards smaller, denser LDL subfractions, a pattern that has previously been associated with cardiometabolic risk [57,58]. Interestingly, a previous study investigating the lipoprotein profiles of children 3 years post-burn injury (aged 4–8 years) also found an elevation in the small dense LDL subfractions [57]. The consistency of this observation across studies suggests that altered LDL particle distribution may represent a persistent feature of post-burn metabolic remodelling. Similar LDL particle distributions have been described in populations with cardiovascular disease and diabetes [15,20], providing context for the observed phenotype but not evidence of equivalent disease risk in this cohort.

**Fig 3:**
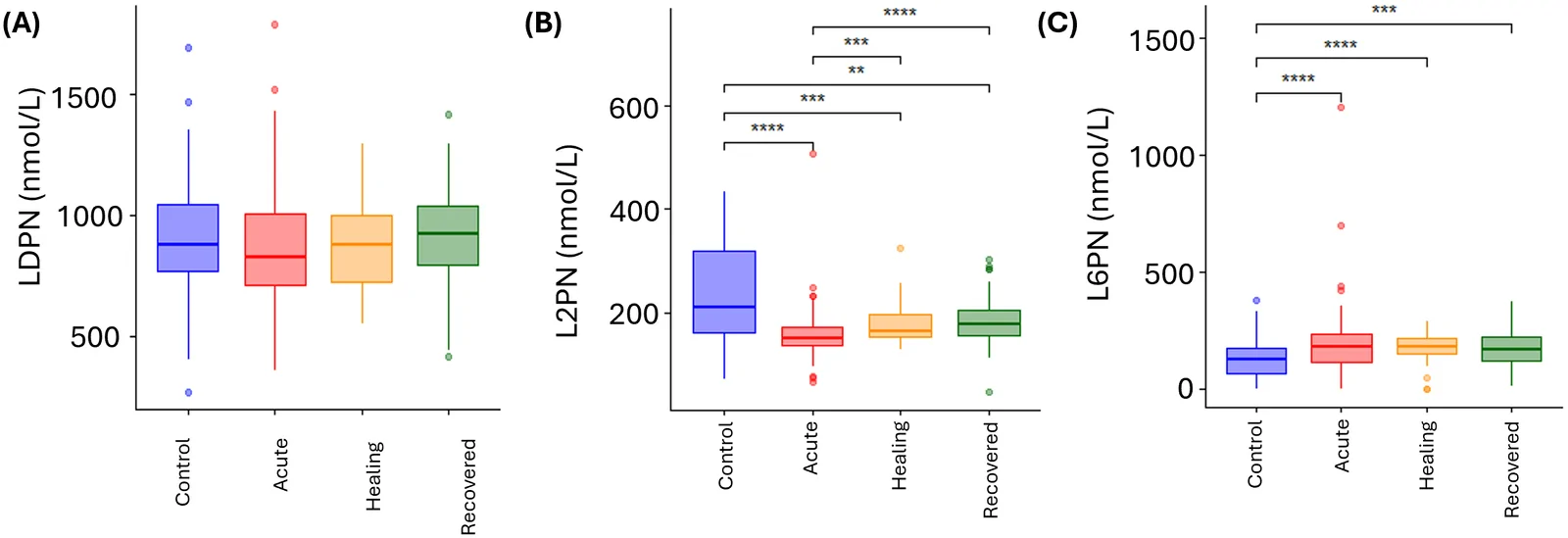
Box plots of LDL particle number for the non-burn controls (blue), acute burn injury (red), healing (yellow) and recovered (green) burns patients. **(A)** total LDL particle number (LDPN), **(B)** LDL subfraction 2 particle number (L2PN), **(C)** LDL subfraction 6 particle number (L6PN). *p < 0.05; **p < 0.01; ***p < 0.001; ****p < 0.0001, determined using the Wilcoxon test.

To further highlight this shift, a comparison of the LDL particle distribution was completed (Fig 4 and S27 Fig in S1 File). The larger more buoyant LDL subfractions (LDL subfractions 1−3) were found to be reduced in the recovered children, from 65.5% to 59.5%, while the smaller less buoyant LDL subfractions (LDL subfractions 4−6) were increased from 34.5% to 40.5%. As seen previously, the strongest changes were observed in the reduction of LDL subfraction 2 from 26.7% to 20.5% of the total LDL composition (adjusted *p*-value = 1.50x10^-7^) and the increase in LDL subfraction 6 from 12.5% to 17.8% of the total LDL composition (adjusted *p*-value = 3.01x10^-4^). Overall, the total LDL concentration remained unchanged between non-burn controls and recovered paediatric patients, but this redistribution towards smaller LDLs is a lipoprotein phenotype that has been associated with cardiovascular and metabolic risk in previous studies [20,58,59].

**Fig 4:**
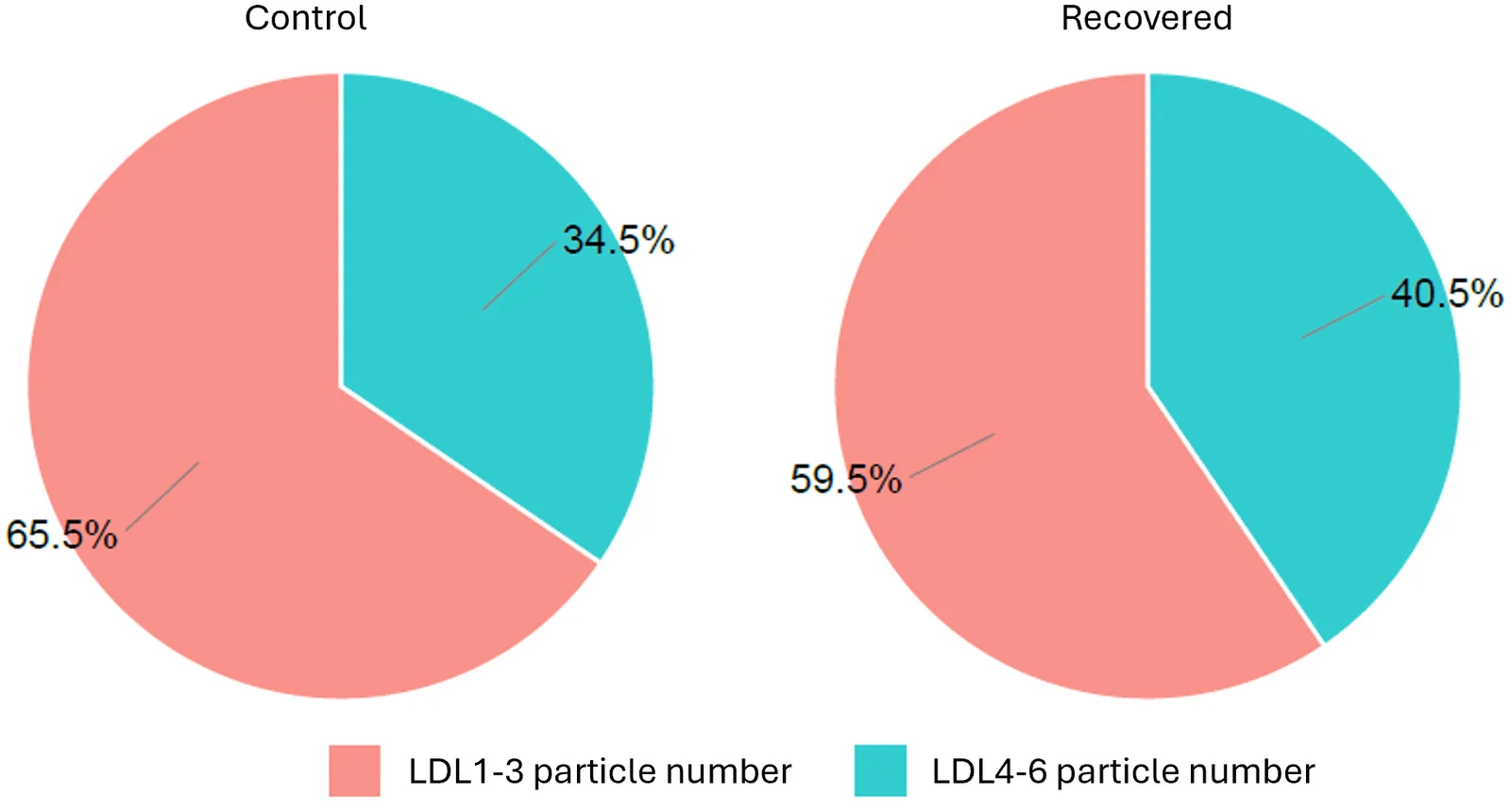
LDL subfraction particle number percentages for the non-burn control and recovered paediatric patients for the larger LDL (LDL 1-3 particle number) and smaller LDL (LDL 4-6 particle number).

The observed changes in HDL in the paediatric burn injury samples also support a phenotypic shift (S10 Fig in S1 File). While total HDL levels were not significantly different between non-burn controls and 1-year post-burn injury (adjusted *p*-value = 0.06), some HDL lipoprotein subfractions were significantly changed between the two groups. HDL subfraction 1 (H1A1), the largest HDL subfraction, was significantly reduced (adjusted *p*-value = 2.90x10^-3^) in the recovered phase in comparison to non-burn controls. Typically, higher levels of HDL have been positively associated with health [19], whereas depletion has been associated with inflammatory conditions, disease or obesity [52].

The reduction in the larger HDL particles and what appears to be normal levels of HDL subfraction 4 represents a lipoprotein phenotype that has previously been described in chronic inflammatory and metabolic conditions in adults. This was previously demonstrated using SPC, the Supramolecular Phospholipid Composite (SPC) NMR peak, where SPC_1_ arises from the PL contained in HDL subfraction 4, and the SPC_2_ signal arises from the PL contained in HDL1–3 [32,53] SPC_1_ remained unperturbed and SPC_2_ reduced in chronic inflammatory states, consistent with the findings in this study that demonstrates a continued chronic inflammatory state in the paediatric patients one year post-burn injury.

*Long-term alterations in lipoprotein composition post-burn injury:* While there were observed particle number changes in VLDL and IDL, the total lipoprotein particle number remained unchanged in the recovered patients compared to non-burn controls (S4A Fig in S1 File). However, there were significant changes in the lipid composition of all main lipoprotein fractions (Fig 5). The VLDL subfractions had a significant reduction in the composition of CE (adjusted *p*-value = 9.38x10^-4^) and PLs (adjusted *p*-value = 1.21x10^-4^) while the percentage of TGs significantly increased (adjusted *p*-value = 9.79x10^-6^) in the recovered participants compared to the non-burn controls. An increase in TG content within VLDL subfractions has previously been associated with cardiometabolic risk [60], although the clinical significance of this compositional alteration in children following burn injury remains unknown. Overall, we observed a reduction in total VLDL in recovered burns patients and the total TG levels were lower in this study in comparison to non-burn controls. The reduced VLDL levels observed could mean a possible decrease in the amount of VLDL produced by the liver or a faster turnover of VLDL in the bloodstream, suggestive of a hypermetabolic state. Human lipoprotein kinetics studies may provide insight into this [56].

**Fig 5:**
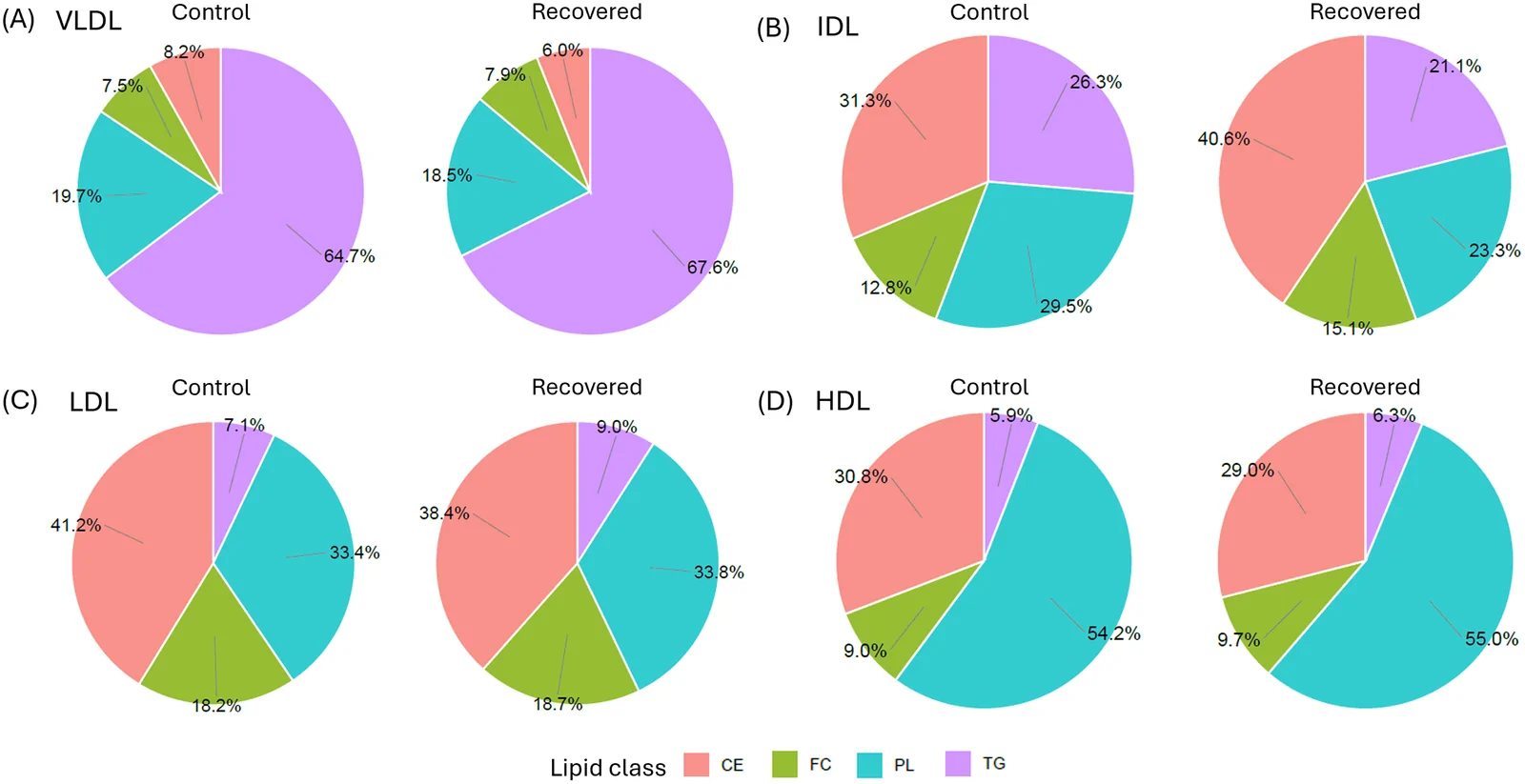
Lipoprotein composition according to the main lipid components of cholesterol ester (CE), free cholesterol (FC), phospholipid (PL) and triglycerides (TG) for the non-burn controls and recovered burn patients for (A) VLDL, (B) IDL, (C) LDL and (D) HDL. Percentage comparisons between lipids and *p*-values can be found in S121 Table in S1 File.

The largest compositional changes were observed in IDLs, which are formed when VLDL particles lose TGs in the bloodstream and thereby decrease in size [58]. In recovered children, the proportion of CE in IDLs increased markedly from 31.3% to 40.6% compared with non-burn controls (adjusted *p*-value = 2.41 × 10⁻^3^). Elevated CE content in IDL has been associated with atherogenic lipoprotein profiles in previous studies, as has been shown to promote arterial plaque formation [61,62]. The recovered group also showed increased FC and reduced PL (adjusted *p*-value = 3.23 × 10⁻^2^). These changes are consistent with changes associated with atherosclerotic risk, since IDL cholesterol enrichment is characteristic of familial hypercholesterolemia and is associated with higher levels of small, dense LDL particles, likely derived from smaller IDL species [63,64]. Notably, higher levels of smaller LDL were also observed in this study. In LDL, PL content did not differ between recovered and control groups. However, CE was significantly reduced in recovered paediatric patients (adjusted *p*-value = 5.51 × 10⁻⁴), while FC and TGs were elevated.

HDL composition showed the opposite pattern to LDL; CE was significantly decreased in recovered children (adjusted *p*-value = 2.69 × 10⁻⁴), whereas FC (adjusted *p*-value = 5.84 × 10⁻^3^) and PL (adjusted *p*-value = 6.30 × 10⁻^3^) were significantly increased. Changes in HDL lipid composition can arise from altered lipid transfer and remodelling between HDL and apoB-containing lipoproteins, as well as changes in hepatic uptake and processing of HDL-derived cholesterol. Increased triglyceride enrichment of HDL has been proposed to alter HDL particle remodelling and cholesterol delivery to the liver [65,66]. Thus, the compositional changes observed in recovered children may reflect altered HDL metabolism during longer-term post-burn metabolic adaptation.

A limitation of this study is that samples were obtained from multiple cohorts, with plasma collected from the Australian control and burn cohorts and serum from the US control cohort. Although identical analytical protocols were used, detailed metadata on collection tube type, storage duration, and freeze–thaw history were not available for all externally sourced samples and therefore could not be included as covariates. However, PCA and OPLS-DA demonstrated no meaningful separation by geographical origin, suggesting that these pre-analytical differences were not a major source of lipoproteomic variation.

An additional limitation is that the present study characterises circulating lipoprotein concentrations and composition but does not directly measure the biological pathways responsible for these changes. Inflammatory cytokines, markers of hepatic lipid metabolism, adipose tissue lipolysis, lipoprotein lipase activity, lipoprotein production and clearance rates, insulin sensitivity and HDL functional measures were not assessed. Consequently, the proposed mechanisms involving inflammation, hepatic lipid handling, adipose tissue mobilisation and lipoprotein remodelling should be considered biologically plausible interpretations rather than mechanisms directly demonstrated by the present study.

A further important consideration is that the lipoprotein alterations identified in this study are surrogate biomarkers rather than direct measures of cardiovascular disease risk. Although several observed patterns, including increased small dense LDL subfractions, altered HDL composition and changes in apolipoprotein ratios, have been associated with cardiovascular and metabolic risk in previous epidemiological studies, the present study did not assess cardiovascular events, vascular function, validated cardiovascular risk scores or other clinical cardiovascular endpoints. Furthermore, inflammatory biomarkers were not available to directly determine whether the persistent lipoprotein alterations reflected ongoing inflammation. Therefore, the observed lipoproteomic differences should be interpreted as evidence of persistent metabolic and lipoprotein perturbation following burn injury.

## Conclusions

This study demonstrates that alterations in the lipoproteome prevail beyond the acute phase following burn injury, with some factors resolving, some persisting and others emerging as part of a new chronic phenotype. In the acute phase, paediatric burn lipoprotein signatures share similarities with those observed in adult burns and inflammatory conditions such as COVID-19, with marked increases in LDL TGs and ABA1, and reductions in HDL subfractions. Beyond 1-year post-burn, total VLDL and IDL concentrations were significantly reduced, while total LDL and HDL levels remained unchanged from non-burn controls. In addition, a shift in LDL subfraction distribution was evident, characterised by a reduction in larger LDL subfractions and a higher proportion of smaller LDL subfractions in comparison to non-burn controls, a lipoprotein pattern previously associated with increased cardiometabolic risk. Furthermore, all major lipoprotein classes exhibited compositional changes, including an increased TG content in VLDL and HDL and a rise in CE levels in IDL, which have previously been associated with altered metabolic and cardiovascular risk profiles but whose clinical significance following paediatric burn injury remains uncertain.

These findings highlight the limitations of traditional blood chemistry assessments in post-burn clinical management. While routine lipid panels may suggest recovery after 1 year, underlying lipoproteomic alterations persist. A more comprehensive lipoprotein analysis incorporating mechanistic and pathway level interpretation alongside inflammatory biomarkers, validated cardiovascular risk assessments, vascular measures and longitudinal clinical outcomes is warranted to better understand the long-term metabolic implications of paediatric burn trauma. Such approaches would help elucidate the underlying biological processes driving the observed alterations in lipid metabolism and determine whether these persistent lipoproteomic changes are associated with future cardiovascular or metabolic disease.

## Supporting information

S1 File Supplementary tables 1–121 and figures 1–27. (DOCX)
