# Supplementary material for: Blood lipoprotein remodelling after non-severe paediatric burns: A longitudinal analysis beyond the acute phase
Source: PLoS One. 2026 Nov 12;21(11):e0362177. doi: 10.1371/journal.pone.0362177 (PMC13759082; doi:10.1371/journal.pone.0362177)
Supplement: Supplementary file 1 [file pone.0362177.s001.docx]

Blood lipoprotein remodelling after non-severe paediatric burns: A longitudinal analysis beyond the acute phase

Monique J Ryan^1^, Chloe Scott^1^, Nathan G Lawler^1,2^, Reika Masuda^1^, Jacqueline A Davis^3^, Nina D’Vaz^3^, Lael M Yonker^4,5^, Maureen M Leonard^4,5^, Alessio Fasano^4,5^, Luke Whiley^1,6,7,8^, Jeremy K Nicholson^9,10^, Elaine Holmes^10,11^, Fiona M Wood^12,13,14*^, Samantha Lodge^1,15,16*^

*^1^Centre for Computational and Systems Medicine, Health Futures Institute, Murdoch University, Harry Perkins Building, Perth, WA6150, Australia.*

*^2^School of Allied Health (Exercise Science), Murdoch University, Perth, WA6150, Australia.*

*^3^The Kids Research Institute Australia, Perth, WA 6009, Australia.*

*^4^Massachusetts General Hospital, Mucosal Immunology and Biology Research Center, Department of Pediatrics, Boston, MA, USA.*

*^5^Harvard Medical School, Boston, MA, USA.*

*^6^School of Diagnostic and Therapeutic Sciences, Curtin University, Perth, WA 6102, Australia.*

*^7^Curtin Medical Research Institute, Curtin University, Perth, WA 6102, Australia.*

*^8^Dementia Centre of Excellence, enAble Institute, Curtin University, Perth, WA 6102, Australia*

*^9^Institute of Global Health Innovation, Faculty of Medicine, Imperial College London, London SW7 2NA, UK.*

*^10^School of Medicine, The Hong Kong University of Science and Technology, Hong Kong, China.*

*^11^Section of Nutrition, Department of Metabolism, Digestion and Reproduction, Faculty of Medicine, Imperial College London, Sir Alexander Fleming Building, South Kensington, London SW7 2AZ, UK.*

*^12^Burn Injury Research Unit, School of Biomedical Sciences, University of Western Australia, Perth, WA 6009, Australia.*

*^13^Fiona Wood Foundation, Perth, WA 6150, Australia.*

*^14^Burns Service of Western Australia, WA Department of Health, Perth, WA 6150, Australia.*

*^15^School of Medical, Molecular and Forensic Sciences, Murdoch University, Murdoch, Perth, WA6150, Australia.*

*^16^School of Biomedical Sciences, University of Western Australia, Perth, WA 6009, Australia.*

Table of contents

| **Description** | **Page** |
| --- | --- |
| Table S1 – Cohort demographics | S8 |
| Table S2 – Annotation of the keys used by the Bruker IVDr Lipoprotein Subclass Analysis (B.I-LISA^TM^) method | S9 |
| Figure S1 – PCA scores plot of the controls from Australia (red) and those from the USA (blue) after age correction. | S11 |
| Table S3 – OPLS loadings, Cliff’s delta and adjusted *p*-values of the lipoproteins for the comparison of the non-burn controls and acute burns patients. | S12 |
| Figure S2 – Multivariate model performance evaluation and classification accuracy of the OPLS-DA model for the non-burn control vs acute burn injury (Figure 1). | S15 |
| Table S4 – OPLS loadings, Cliff’s delta and adjusted *p-*values of the lipoproteins for the comparison of the non-burn controls and healing patients. | S16 |
| Table S5 – OPLS loadings, Cliff’s delta and adjusted *p*-values of the lipoproteins for the comparison of the non-burn controls and recovered patients. | S19 |
| Figure S3 – Box plots of the main lipoprotein parameter concentrations and calculated ratios for the non-burn controls, acute burn injury, healing and recovered burns patients. | S22 |
| Table S6 – Group comparisons of total triglyceride (TPTG). | S23 |
| Table S7 – Group comparisons of total cholesterol (TPCH). | S23 |
| Table S8 – Group comparisons of LDL cholesterol (LDCH). | S23 |
| Table S9 – Group comparisons of HDL cholesterol (HDCH). | S23 |
| Table S10 – Group comparisons of total apolipoprotein A1 (TPA1). | S24 |
| Table S11 – Group comparisons of total apolipoprotein A2 (TPA2). | S24 |
| Table S12 – Group comparisons of total apolipoprotein B100 (TPAB). | S24 |
| Table S13 – Group comparisons of LDL-cholesterol/HDL-cholesterol (LDHD). | S24 |
| Table S14 – Group comparisons of Apolipoprotein B100/Apolipoprotein A1 (ABA1). | S25 |
| Figure S4 – Box plots of the total concentration of Apolipoprotein B100 carrying particles for the non-burn controls, acute burn injury, healing and recovered burns patients. | S25 |
| Table S15 – Group comparisons of total particle number (TBPN). | S26 |
| Table S16 – Group comparisons of very low-density particle number (VLPN). | S26 |
| Table S17 – Group comparisons of intermediate density particle number (IDPN). | S26 |
| Table S18 – Group comparisons of low-density particle number (LDPN). | S26 |
| Figure S5 – Box plots of the total concentration of LDL subfractions for the non-burn controls, acute burn injury, healing and recovered burns patients. | S27 |
| Table S19 – Group comparisons of LDL subfraction 1 particle number (L1PN). | S27 |
| Table S20 – Group comparisons of LDL subfraction 2 particle number (L2PN). | S28 |
| Table S21 – Group comparisons of LDL subfraction 3 particle number (L3PN). | S28 |
| Table S22 – Group comparisons of LDL subfraction 4 particle number (L4PN). | S28 |
| Table S23 – Group comparisons of LDL subfraction 5 particle number (L5PN). | S28 |
| Table S24 – Group comparisons of LDL subfraction 6 particle number (L6PN). | S29 |
| Figure S6 – Box plots of the total concentration of triglycerides in the main lipoprotein fractions for the non-burn controls, acute burn injury, healing and recovered burns patients. | S29 |
| Table S25 – Group comparisons of VLDL triglyceride (VLTG). | S30 |
| Table S26 – Group comparisons of IDL triglyceride (IDTG). | S30 |
| Table S27 – Group comparisons of LDL triglyceride (LDTG). | S30 |
| Table S28 – Group comparisons of HDL triglyceride (HDTG) | S30 |
| Figure S7 – Box plots of the total concentration of cholesterol in the main lipoprotein fractions for the non-burn controls, acute burn injury, healing and recovered burns patients. | S31 |
| Table S29 – Group comparisons of VLDL cholesterol (VLCH). | S31 |
| Table S30 – Group comparisons of IDL cholesterol (IDCH). | S32 |
| Table S31 – Group comparisons of LDL cholesterol (LDCH). | S32 |
| Table S32 – Group comparisons of HDL cholesterol (HDCH). | S32 |
| Figure S8 – Box plots of the total concentration of free cholesterol in the main lipoprotein fractions for the non-burn controls, acute burn injury, healing and recovered burns patients. | S33 |
| Table 33 – Group comparisons of VLDL free cholesterol (VLFC). | S33 |
| Table 34 – Group comparisons of IDL free cholesterol (IDFC). | S34 |
| Table 35 – Group comparisons of LDL free cholesterol (LDFC). | S34 |
| Table 36 – Group comparisons of HDL free cholesterol (HDFC). | S34 |
| Figure S9 – Box plots of the total concentration of phospholipid in the main lipoprotein fractions for the non-burn controls, acute burn injury, healing and recovered burns patients. | S35 |
| Table 37 – Group comparisons of VLDL phospholipid (VLPL). | S35 |
| Table 38 – Group comparisons of IDL phospholipid (IDPL). | S36 |
| Table 39 – Group comparisons of LDL phospholipid (LDPL). | S36 |
| Table 40 – Group comparisons of HDL phospholipid (HDPL). | S36 |
| Figure S10 – Box plots of the total concentration of Apolipoprotein A1 and Apolipoprotein B100 in the main lipoprotein fractions for the non-burn controls, acute burn injury, healing and recovered burns patients. | S37 |
| Table S41 – Group comparisons of HDL Apo-A1 (HDA1). | S37 |
| Table S42 – Group comparisons of HDL Apo-A2 (HDA2). | S38 |
| Table S43 – Group comparisons of VLDL Apo-B100 (VLAB). | S38 |
| Table S44 – Group comparisons of IDL Apo-B100 (IDAB). | S38 |
| Table S45 – Group comparisons of LDL Apo-B100 (LDAB). | S38 |
| Figure S11 – Box plots of the triglyceride concentration in the VLDL subfractions for the non-burn controls, acute burn injury, healing and recovered burns patients. | S39 |
| Table S46 – Group comparisons of VLDL subfraction 1 triglyceride (V1TG). | S39 |
| Table S47 – Group comparisons of VLDL subfraction 2 triglyceride (V2TG). | S40 |
| Table S48 – Group comparisons of VLDL subfraction 3 triglyceride (V3TG). | S40 |
| Table S49 – Group comparisons of VLDL subfraction 4 triglyceride (V4TG). | S40 |
| Table S50 – Group comparisons of VLDL subfraction 5 triglyceride (V5TG). | S40 |
| Figure S12 – Box plots of the cholesterol concentration in the VLDL subfractions for the non-burn controls, acute burn injury, healing and recovered burns patients. | S41 |
| Table S51 – Group comparisons of VLDL subfraction 1 cholesterol (V1CH). | S41 |
| Table S52 – Group comparisons of VLDL subfraction 2 cholesterol (V2CH). | S42 |
| Table S53 – Group comparisons of VLDL subfraction 3 cholesterol (V3CH). | S42 |
| Table S54 – Group comparisons of VLDL subfraction 4 cholesterol (V4CH). | S42 |
| Table S55 – Group comparisons of VLDL subfraction 5 cholesterol (V5CH). | S42 |
| Figure S13 – Box plots of the free cholesterol concentration in the VLDL subfractions for the non-burn controls, acute burn injury, healing and recovered burns patients. | S43 |
| Table S56 – Group comparisons of VLDL subfraction 1 free cholesterol (V1FC). | S43 |
| Table S57 – Group comparisons of VLDL subfraction 2 free cholesterol (V2FC). | S44 |
| Table S58 – Group comparisons of VLDL subfraction 3 free cholesterol (V3FC). | S44 |
| Table S59 – Group comparisons of VLDL subfraction 4 free cholesterol (V4FC). | S44 |
| Table S60 – Group comparisons of VLDL subfraction 5 free cholesterol (V5FC). | S44 |
| Figure S14 – Box plots of the phospholipid concentration in the VLDL subfractions for the non-burn controls, acute burn injury, healing and recovered burns patients. | S45 |
| Table S61 – Group comparisons of VLPL subfraction 1 phospholipid (V1PL). | S45 |
| Table S62 – Group comparisons of VLPL subfraction 2 phospholipid (V2PL). | S46 |
| Table S63 – Group comparisons of VLPL subfraction 3 phospholipid (V3PL). | S46 |
| Table S64 – Group comparisons of VLPL subfraction 4 phospholipid (V4PL). | S46 |
| Table S65 – Group comparisons of VLPL subfraction 5 phospholipid (V5PL). | S46 |
| Figure S15 – Box plots of the triglyceride concentration in the LDL subfractions for the non-burn controls, acute burn injury, healing and recovered burns patients. | S47 |
| Table S66 – Group comparisons of LDL subfraction 1 triglyceride (L1TG). | S47 |
| Table S67 – Group comparisons of LDL subfraction 2 triglyceride (L2TG). | S48 |
| Table S68 – Group comparisons of LDL subfraction 3 triglyceride (L3TG). | S48 |
| Table S69 – Group comparisons of LDL subfraction 4 triglyceride (L4TG). | S48 |
| Table S70 – Group comparisons of LDL subfraction 5 triglyceride (L5TG). | S48 |
| Table S71 – Group comparisons of LDL subfraction 6 triglyceride (L6TG). | S49 |
| Figure S16 – Box plots of the cholesterol concentration in the LDL subfractions for the non-burn controls, acute burn injury, healing and recovered burns patients. | S49 |
| Table S72 – Group comparisons of LDL subfraction 1 cholesterol (L1CH). | S50 |
| Table S73 – Group comparisons of LDL subfraction 2 cholesterol (L2CH). | S50 |
| Table S74 – Group comparisons of LDL subfraction 3 cholesterol (L3CH). | S50 |
| Table S75 – Group comparisons of LDL subfraction 4 cholesterol (L4CH). | S50 |
| Table S76 – Group comparisons of LDL subfraction 5 cholesterol (L5CH). | S51 |
| Table S77– Group comparisons of LDL subfraction 6 cholesterol (L6CH). | S51 |
| Figure S17 – Box plots of the free cholesterol concentration in the LDL subfractions for the non-burn controls, acute burn injury, healing and recovered burns patients. | S52 |
| Table S78 – Group comparisons of LDL subfraction 1 free cholesterol (L1FC). | S52 |
| Table S79 – Group comparisons of LDL subfraction 2 free cholesterol (L2FC). | S53 |
| Table S80 – Group comparisons of LDL subfraction 3 free cholesterol (L3FC). | S53 |
| Table S81 – Group comparisons of LDL subfraction 4 free cholesterol (L4FC). | S53 |
| Table S82 – Group comparisons of LDL subfraction 5 free cholesterol (L5FC). | S53 |
| Table S83 – Group comparisons of LDL subfraction 3 free cholesterol (L6FC). | S54 |
| Figure S18 – Box plots of the phospholipid concentration in the LDL subfractions for the non-burn controls, acute burn injury, healing and recovered burns patients. | S54 |
| Table S84 – Group comparisons of LDL subfraction 1 phospholipid (L1PL). | S55 |
| Table S85 – Group comparisons of LDL subfraction 2 phospholipid (L2PL). | S55 |
| Table S86 – Group comparisons of LDL subfraction 3 phospholipid (L3PL). | S55 |
| Table S87– Group comparisons of LDL subfraction 4 phospholipid (L4PL). | S55 |
| Table S88 – Group comparisons of LDL subfraction 5 phospholipid (L5PL). | S56 |
| Table S89 – Group comparisons of LDL subfraction 6 phospholipid (L6PL). | S56 |
| Figure S19 – Box plots of the Apolipoprotein B100 concentration in the LDL subfractions for the non-burn controls, acute burn injury, healing and recovered burns patients. | S57 |
| Table S90 – Group comparisons of LDL subfraction 1 Apo-B (L1AB). | S57 |
| Table S91 – Group comparisons of LDL subfraction 2 Apo-B (L2AB). | S58 |
| Table S92 – Group comparisons of LDL subfraction 3 Apo-B (L3AB). | S58 |
| Table S93 – Group comparisons of LDL subfraction 4 Apo-B (L4AB). | S58 |
| Table S94 – Group comparisons of LDL subfraction 5 Apo-B (L5AB). | S58 |
| Table S95 – Group comparisons of LDL subfraction 6 Apo-B (L6AB). | S59 |
| Figure S20 – Box plots of the triglyceride concentration in the HDL subfractions for the non-burn controls, acute burn injury, healing and recovered burns patients. | S59 |
| Table S96 – Group comparisons of HDL subfraction 1 triglyceride (H1TG). | S60 |
| Table S97 – Group comparisons of HDL subfraction 2 triglyceride (H2TG). | S60 |
| Table S98 – Group comparisons of HDL subfraction 3 triglyceride (H3TG). | S60 |
| Table S99 – Group comparisons of HDL subfraction 4 triglyceride (H4TG). | S60 |
| Figure S21 – Box plots of the cholesterol concentration in the HDL subfractions for the non-burn controls, acute burn injury, healing and recovered burns patients. | S61 |
| Table S100 – Group comparisons of HDL subfraction 1 cholesterol (H1CH). | S61 |
| Table S101 – Group comparisons of HDL subfraction 2 cholesterol (H2CH). | S62 |
| Table S102 – Group comparisons of HDL subfraction 3 cholesterol (H3CH). | S62 |
| Table S103 – Group comparisons of HDL subfraction 4 cholesterol (H4CH). | S62 |
| Figure S22 – Box plots of the free cholesterol concentration in the HDL subfractions for the non-burn controls, acute burn injury, healing and recovered burns patients. | S63 |
| Table S104 – Group comparisons of HDL subfraction 1 free cholesterol (H1FC). | S63 |
| Table S105 – Group comparisons of HDL subfraction 2 free cholesterol (H2FC). | S64 |
| Table S106 – Group comparisons of HDL subfraction 3 free cholesterol (H3FC). | S64 |
| Table S107 – Group comparisons of HDL subfraction 4 free cholesterol (H4FC). | S64 |
| Figure S23 – Box plots of the phospholipid concentration in the HDL subfractions for the non-burn controls, acute burn injury, healing and recovered burns patients. | S65 |
| Table S108 – Group comparisons of HDL subfraction 1 phospholipid (H1PL). | S65 |
| Table S109 – Group comparisons of HDL subfraction 2 phospholipid (H2PL). | S66 |
| Table S110 – Group comparisons of HDL subfraction 3 phospholipid (H3PL). | S66 |
| Table S111 – Group comparisons of HDL subfraction 4 phospholipid (H4PL). | S66 |
| Figure S24 – Box plots of the Apolipoprotein A1 concentration in the HDL subfractions for the non-burn controls, acute burn injury, healing and recovered burns patients. | S67 |
| Table S112 – Group comparisons of HDL subfraction 1 Apo-A1 (H1A1). | S67 |
| Table S113 – Group comparisons of HDL subfraction 2 Apo-A1 (H2A1). | S68 |
| Table S114 – Group comparisons of HDL subfraction 3 Apo-A1 (H3A1). | S68 |
| Table S115 – Group comparisons of HDL subfraction 4 Apo-A1 (H4A1). | S68 |
| Figure S25 – Box plots of the Apolipoprotein A2 concentration in the HDL subfractions for the non-burn controls, acute burn injury, healing and recovered burns patients. | S69 |
| Table S116 – Group comparisons of HDL subfraction 1 Apo-A2 (H1A2). | S69 |
| Table S117 – Group comparisons of HDL subfraction 2 Apo-A2 (H2A2). | S70 |
| Table S118 – Group comparisons of HDL subfraction 3 Apo-A2 (H3A2). | S70 |
| Table S119 – Group comparisons of HDL subfraction 4 Apo-A2 (H4A2). | S70 |
| Figure S26 – Validation of the OPLS-DA model by permutation testing (Figure 2). | S71 |
| Figure S27 – LDL particle numbers for the control and recovered patients. | S72 |
| Table S120 – Percentage composition of the LDL by subfraction size. | S72 |
| Table S121 – Percentage composition of lipids within the main lipoprotein fractions, VLDL, IDL, LDL and HDL. | S73 |

**Table S1 – Cohort demographics**

|  | **Non burn Controls (n=103)** | **Paediatric burns patients (n=142)** |
| --- | --- | --- |
| Sex, Male | 52 (53.06%) | 80 (56.34%) |
| Age at first blood collection, years* [SD] | 3.00 [±4.64] | 3.59 [±4.54] |
| Total burn surface area* (%)  [SD] | - | 2.00 [±5.85] |
| Burn type  Chemical  Contact  Electrical  Flame  Friction  Scald | - | 3 (0.02%)  39 (27.46%)  3 (0.02%)  13 (9.15%)  18 (12.68%)  66 (46.48%) |
| Surgery required | - | 123 (86.62%) |
| Surgery type  Recell  Split thickness skin graft  Split skin graft and recell  Other | - | 72 (58.54%)  19 (15.45%)  25 (20.33%)  7 (5.70%) |
| Days to re-epithelisation |  | 21.00 [±23.59] |

*Group median

## **Quantification of Plasma Lipoproteins**

A total of 112 lipoprotein parameters were quantified based on 1D NMR experiments as part of Bruker’s IVDr experiment suite for blood plasma (Table S4). This approach is termed Bruker’s IVDr lipoprotein class and subclass Analysis (B.I.-LISA™) and lipid analytes include cholesterol, free cholesterol, phospholipids, triglycerides, apolipoproteins A1/A2/B100 and ratio B100/A1, in total plasma concentration and resolved for main lipoprotein classes and subclasses. Main classes of plasma-lipoproteins were defined as: high-density lipoprotein (HDL, density 1.063–1.210 kg/L), intermediate-density lipoprotein (IDL, density 1.006–1.019 kg/L) low-density lipoprotein (LDL, density 1.019–1.063 kg/L) and very low-density lipoprotein (VLDL, 0.950–1.006 kg/L). The main lipoprotein classes HDL, LDL, VLDL were further divided into different lipoprotein sub-classes:  (LDL-1: 1.019–1.031 kg/L, LDL-2: 1.031–1.034 kg/L, LDL-3: 1.034–1.037 kg/L, LDL-4: 1.037–1.040 kg/L, LDL-5: 1.040–1.044 kg/L, LDL-6: 1.044–1.063 kg/L), and the HDL sub-fractions into four density classes (HDL-1 1.063–1.100 kg/L, HDL-2 1.100–1.125 kg/L, HDL-3 1.125–1.175 kg/L, and HDL-4 1.175–1.210 kg/L), the VLDL sub-fractions divided into 5 density classes.

**Table S2 - Annotation of the keys used by the Bruker IVDr Lipoprotein Subclass Analysis (B.I.-LISA ^TM^) method.** Abbreviations: LDL – low-density lipoprotein; HDL – high-density lipoprotein; VLDL – very low-density lipoprotein; IDL – intermediate-density lipoprotein.

| **Key** | **Class/subclass** | **Compound** | **Concentration unit** |
| --- | --- | --- | --- |
| TPTG | Total Plasma | Triglycerides | mg/dL |
| TPCH | Total Plasma | Cholesterol | mg/dL |
| TPA1 | Total Plasma | Apolipoprotein-A1 | mg/dL |
| TPA2 | Total Plasma | Apolipoprotein-A2 | mg/dL |
| TPAB | Total Plasma | Apolipoprotein-B100 | mg/dL |
| LDHD | Ratio LDL and HDL Cholesterol | LDL Cholesterol / HDL Cholesterol | -/- |
| ABA1 | Ratio of Apolipoproteins B100 and A1 | Apolipoprotein-B100 / Apolipoprotein-A1 | -/- |
| TBPN | Apolipoprotein-B100 carrying particles | Particle Number | nmol/L |
| VLPN | VLDL | Particle Number | nmol/L |
| IDPN | IDL | Particle Number | nmol/L |
| LDPN | LDL | Particle Number | nmol/L |
| L1PN | LDL-1 | Particle Number | nmol/L |
| L2PN | LDL-2 | Particle Number | nmol/L |
| L3PN | LDL-3 | Particle Number | nmol/L |
| L4PN | LDL-4 | Particle Number | nmol/L |
| L5PN | LDL-5 | Particle Number | nmol/L |
| L6PN | LDL-6 | Particle Number | nmol/L |
| VLTG | VLDL Class | Triglycerides | mg/dL |
| IDTG | IDL Class | Triglycerides | mg/dL |
| LDTG | LDL Class | Triglycerides | mg/dL |
| HDTG | HDL Class | Triglycerides | mg/dL |
| VLCH | VLDL Class | Cholesterol | mg/dL |
| IDCH | IDL Class | Cholesterol | mg/dL |
| LDCH | LDL Class | Cholesterol | mg/dL |
| HDCH | HDL Class | Cholesterol | mg/dL |
| VLFC | VLDL Class | Free Cholesterol | mg/dL |
| IDFC | IDL Class | Free Cholesterol | mg/dL |
| LDFC | LDL Class | Free Cholesterol | mg/dL |
| HDFC | HDL Class | Free Cholesterol | mg/dL |
| VLPL | VLDL Class | Phospholipids | mg/dL |
| IDPL | IDL Class | Phospholipids | mg/dL |
| LDPL | LDL Class | Phospholipids | mg/dL |
| HDPL | HDL Class | Phospholipids | mg/dL |
| HDA1 | HDL Class | Apolipoprotein-A1 | mg/dL |
| HDA2 | HDL Class | Apolipoprotein-A2 | mg/dL |
| VLAB | VLDL Class | Apolipoprotein-B100 | mg/dL |
| IDAB | IDL Class | Apolipoprotein-B100 | mg/dL |
| LDAB | LDL Class | Apolipoprotein-B100 | mg/dL |
| V1TG | VLDL-1 Subclass | Triglycerides | mg/dL |
| V2TG | VLDL-2 Subclass | Triglycerides | mg/dL |
| V3TG | VLDL-3 Subclass | Triglycerides | mg/dL |
| V4TG | VLDL-4 Subclass | Triglycerides | mg/dL |
| V5TG | VLDL-5 Subclass | Triglycerides | mg/dL |
| V1CH | VLDL-1 Subclass | Cholesterol | mg/dL |
| V2CH | VLDL-2 Subclass | Cholesterol | mg/dL |
| V3CH | VLDL-3 Subclass | Cholesterol | mg/dL |
| V4CH | VLDL-4 Subclass | Cholesterol | mg/dL |
| V5CH | VLDL-5 Subclass | Cholesterol | mg/dL |
| V1FC | VLDL-1 Subclass | Free Cholesterol | mg/dL |
| V2FC | VLDL-2 Subclass | Free Cholesterol | mg/dL |
| V3FC | VLDL-3 Subclass | Free Cholesterol | mg/dL |
| V4FC | VLDL-4 Subclass | Free Cholesterol | mg/dL |
| V5FC | VLDL-5 Subclass | Free Cholesterol | mg/dL |
| V1PL | VLDL-1 Subclass | Phospholipids | mg/dL |
| V2PL | VLDL-2 Subclass | Phospholipids | mg/dL |
| V3PL | VLDL-3 Subclass | Phospholipids | mg/dL |
| V4PL | VLDL-4 Subclass | Phospholipids | mg/dL |
| V5PL | VLDL-5 Subclass | Phospholipids | mg/dL |
| L1TG | LDL-1 Subclass | Triglycerides | mg/dL |
| L2TG | LDL-2 Subclass | Triglycerides | mg/dL |
| L3TG | LDL-3 Subclass | Triglycerides | mg/dL |
| L4TG | LDL-4 Subclass | Triglycerides | mg/dL |
| L5TG | LDL-5 Subclass | Triglycerides | mg/dL |
| L6TG | LDL-6 Subclass | Triglycerides | mg/dL |
| L1CH | LDL-1 Subclass | Cholesterol | mg/dL |
| L2CH | LDL-2 Subclass | Cholesterol | mg/dL |
| L3CH | LDL-3 Subclass | Cholesterol | mg/dL |
| L4CH | LDL-4 Subclass | Cholesterol | mg/dL |
| L5CH | LDL-5 Subclass | Cholesterol | mg/dL |
| L6CH | LDL-6 Subclass | Cholesterol | mg/dL |
| L1FC | LDL-1 Subclass | Free Cholesterol | mg/dL |
| L2FC | LDL-2 Subclass | Free Cholesterol | mg/dL |
| L3FC | LDL-3 Subclass | Free Cholesterol | mg/dL |
| L4FC | LDL-4 Subclass | Free Cholesterol | mg/dL |
| L5FC | LDL-5 Subclass | Free Cholesterol | mg/dL |
| L6FC | LDL-6 Subclass | Free Cholesterol | mg/dL |
| L1PL | LDL-1 Subclass | Phospholipids | mg/dL |
| L2PL | LDL-2 Subclass | Phospholipids | mg/dL |
| L3PL | LDL-3 Subclass | Phospholipids | mg/dL |
| L4PL | LDL-4 Subclass | Phospholipids | mg/dL |
| L5PL | LDL-5 Subclass | Phospholipids | mg/dL |
| L6PL | LDL-6 Subclass | Phospholipids | mg/dL |
| L1AB | LDL-1 Subclass | Apolipoprotein-B100 | mg/dL |
| L2AB | LDL-2 Subclass | Apolipoprotein-B100 | mg/dL |
| L3AB | LDL-3 Subclass | Apolipoprotein-B100 | mg/dL |
| L4AB | LDL-4 Subclass | Apolipoprotein-B100 | mg/dL |
| L5AB | LDL-5 Subclass | Apolipoprotein-B100 | mg/dL |
| L6AB | LDL-6 Subclass | Apolipoprotein-B100 | mg/dL |
| H1TG | HDL-1 Subclass | Triglycerides | mg/dL |
| H2TG | HDL-2 Subclass | Triglycerides | mg/dL |
| H3TG | HDL-3 Subclass | Triglycerides | mg/dL |
| H4TG | HDL-4 Subclass | Triglycerides | mg/dL |
| H1CH | HDL-1 Subclass | Cholesterol | mg/dL |
| H2CH | HDL-2 Subclass | Cholesterol | mg/dL |
| H3CH | HDL-3 Subclass | Cholesterol | mg/dL |
| H4CH | HDL-4 Subclass | Cholesterol | mg/dL |
| H1FC | HDL-1 Subclass | Free Cholesterol | mg/dL |
| H2FC | HDL-2 Subclass | Free Cholesterol | mg/dL |
| H3FC | HDL-3 Subclass | Free Cholesterol | mg/dL |
| H4FC | HDL-4 Subclass | Free Cholesterol | mg/dL |
| H1PL | HDL-1 Subclass | Phospholipids | mg/dL |
| H2PL | HDL-2 Subclass | Phospholipids | mg/dL |
| H3PL | HDL-3 Subclass | Phospholipids | mg/dL |
| H4PL | HDL-4 Subclass | Phospholipids | mg/dL |
| H1A1 | HDL-1 Subclass | Apolipoprotein-A1 | mg/dL |
| H2A1 | HDL-2 Subclass | Apolipoprotein-A1 | mg/dL |
| H3A1 | HDL-3 Subclass | Apolipoprotein-A1 | mg/dL |
| H4A1 | HDL-4 Subclass | Apolipoprotein-A1 | mg/dL |
| H1A2 | HDL-1 Subclass | Apolipoprotein-A2 | mg/dL |
| H2A2 | HDL-2 Subclass | Apolipoprotein-A2 | mg/dL |
| H3A2 | HDL-3 Subclass | Apolipoprotein-A2 | mg/dL |
| H4A2 | HDL-4 Subclass | Apolipoprotein-A2 | mg/dL |

**Figure S1 – PCA scores plot of the controls from Australia (red) and those from the USA (blue) after age correction.**

**Table S3 - OPLS loadings, Cliff’s delta and adjusted *p*-values of the lipoproteins for the comparison of the non-burn controls and acute burns patients.**

| **Lipoprotein** | **OPLS loadings** | | **Cliff’s delta** | | **Adjusted *p*-value** | |
| --- | --- | --- | --- | --- | --- | --- |
| H3A2 | | 0.176 | | -0.70 | | 6.48x10^-15^ |
| H2A2 | | 0.184 | | -0.66 | | 7.83x10^-15^ |
| L1TG | | 0.172 | | 0.66 | | 2.12x10^-13^ |
| IDPL | | 0.168 | | -0.64 | | 6.43x10^-13^ |
| H2FC | | 0.176 | | -0.62 | | 4.02x10^-12^ |
| H1A1 | | 0.169 | | -0.61 | | 7.41x10^-12^ |
| HDA1 | | 0.165 | | -0.61 | | 1.16x10^-11^ |
| TPA2 | | 0.169 | | -0.60 | | 1.86x10^-11^ |
| H2CH | | 0.184 | | -0.59 | | 4.12x10^-11^ |
| H1FC | | 0.157 | | -0.58 | | 7.05x10^-11^ |
| H1A2 | | 0.156 | | -0.58 | | 1.08x10^-10^ |
| L6TG | | 0.131 | | 0.57 | | 2.00x10^-10^ |
| HDPL | | 0.159 | | -0.57 | | 2.34x10^-10^ |
| TPA1 | | 0.152 | | -0.56 | | 4.30x10^-10^ |
| L2CH | | 0.160 | | -0.56 | | 4.44x10^-10^ |
| HDA2 | | 0.160 | | -0.56 | | 5.33x10^-10^ |
| HDCH | | 0.158 | | -0.56 | | 5.67x10^-10^ |
| H2PL | | 0.172 | | -0.55 | | 8.24x10^-10^ |
| LDTG | | 0.139 | | 0.54 | | 2.12x10^-9^ |
| L2FC | | 0.151 | | -0.54 | | 2.21x10^-9^ |
| TPCH | | 0.146 | | -0.53 | | 3.93x10^-9^ |
| L2PL | | 0.154 | | -0.52 | | 6.79x10^-9^ |
| H3CH | | 0.148 | | -0.51 | | 1.09x10^-8^ |
| L2AB | | 0.147 | | -0.50 | | 2.45x10^-8^ |
| L2PN | | 0.147 | | -0.50 | | 2.45x10^-8^ |
| H1PL | | 0.136 | | -0.49 | | 5.22x10^-8^ |
| LDCH | | 0.126 | | -0.47 | | 1.47x10^-7^ |
| H3PL | | 0.138 | | -0.47 | | 1.81x10^-7^ |
| H2A1 | | 0.130 | | -0.44 | | 7.04x10^-7^ |
| H1CH | | 0.124 | | -0.44 | | 9.62x10^-7^ |
| L3TG | | 0.107 | | 0.43 | | 2.03x10^-6^ |
| L3CH | | 0.114 | | -0.41 | | 4.96x10^-6^ |
| H3FC | | 0.122 | | -0.40 | | 8.80x10^-6^ |
| V1FC | | 0.095 | | -0.39 | | 1.49x10^-5^ |
| HDFC | | 0.106 | | -0.37 | | 2.91x10^-5^ |
| LDPL | | 0.101 | | -0.37 | | 3.64x10^-5^ |
| L6AB | | 0.079 | | 0.37 | | 4.01x10^-5^ |
| L6PN | | 0.079 | | 0.37 | | 4.01x10^-5^ |
| ABA1 | | 0.094 | | 0.37 | | 4.29x10^-5^ |
| L3PL | | 0.103 | | -0.36 | | 5.47x10^-5^ |
| H3A1 | | 0.110 | | -0.36 | | 6.52x10^-5^ |
| V5TG | | 0.075 | | 0.36 | | 7.46x10^-5^ |
| V2PL | | 0.087 | | -0.34 | | 1.71x10^-4^ |
| LDFC | | 0.090 | | -0.34 | | 1.79x10^-4^ |
| L4TG | | 0.099 | | 0.33 | | 2.46x10^-4^ |
| V3PL | | 0.085 | | -0.32 | | 3.25x10^-4^ |
| L1CH | | 0.101 | | -0.32 | | 3.81x10^-4^ |
| L1FC | | 0.094 | | -0.30 | | 7.08x10^-4^ |
| L3AB | | 0.087 | | -0.30 | | 7.08x10^-4^ |
| L3PN | | 0.087 | | -0.30 | | 7.13x10^-4^ |
| V5CH | | 0.067 | | 0.28 | | 1.85x10^-3^ |
| L5TG | | 0.083 | | 0.28 | | 1.94x10^-3^ |
| L6CH | | 0.064 | | 0.28 | | 1.95x10^-3^ |
| V2CH | | 0.076 | | -0.26 | | 3.66x10^-3^ |
| V2TG | | 0.067 | | -0.25 | | 5.72x10^-3^ |
| L6PL | | 0.057 | | 0.24 | | 8.59x10^-3^ |
| L3FC | | 0.074 | | -0.23 | | 8.81x10^-2^ |
| L1PL | | 0.073 | | -0.22 | | 1.30x10^-2^ |
| IDFC | | 0.045 | | -0.20 | | 2.50x10^-2^ |
| VLPL | | 0.051 | | -0.20 | | 2.50x10^-2^ |
| L4CH | | 0.044 | | -0.19 | | 3.58x10^-2^ |
| L5CH | | 0.039 | | -0.18 | | 4.56x10^-2^ |
| V5FC | | 0.047 | | 0.17 | | 5.56x10^-2^ |
| V1PL | | 0.054 | | -0.17 | | 5.95x10^-2^ |
| L5PL | | 0.038 | | -0.17 | | 6.06x10^-2^ |
| H4FC | | 0.038 | | 0.15 | | 8.58x10^-2^ |
| V5PL | | 0.044 | | 0.15 | | 9.37x10^-2^ |
| H4TG | | 0.048 | | 0.15 | | 0.10 |
| VLPN | | 0.043 | | 0.15 | | 0.10 |
| VLAB | | 0.043 | | 0.15 | | 0.10 |
| IDCH | | 0.034 | | -0.14 | | 0.12 |
| L6FC | | 0.036 | | 0.14 | | 0.12 |
| L4FC | | 0.027 | | -0.13 | | 0.15 |
| V3CH | | 0.042 | | -0.13 | | 0.16 |
| IDTG | | 0.051 | | -0.11 | | 0.21 |
| H1TG | | 0.031 | | -0.11 | | 0.21 |
| V4FC | | 0.020 | | -0.10 | | 0.24 |
| L4PL | | 0.017 | | -0.10 | | 0.25 |
| H4A2 | | 0.032 | | -0.10 | | 0.26 |
| LDAB | | 0.018 | | -0.09 | | 0.30 |
| LDPN | | 0.019 | | -0.09 | | 0.30 |
| H4CH | | 0.030 | | -0.09 | | 0.31 |
| V3TG | | 0.029 | | -0.09 | | 0.31 |
| V4TG | | 0.028 | | 0.09 | | 0.32 |
| L4PN | | 0.011 | | -0.09 | | 0.34 |
| L4AB | | 0.011 | | -0.09 | | 0.34 |
| H2TG | | 0.033 | | -0.08 | | 0.40 |
| V3FC | | 0.028 | | -0.07 | | 0.41 |
| H4PL | | 0.024 | | -0.07 | | 0.43 |
| V2FC | | 0.038 | | -0.07 | | 0.45 |
| VLCH | | 0.025 | | -0.06 | | 0.49 |
| VLTG | | 0.031 | | -0.06 | | 0.50 |
| V4CH | | 0.013 | | 0.06 | | 0.50 |
| L1AB | | 0.027 | | -0.06 | | 0.51 |
| L1PN | | 0.027 | | -0.06 | | 0.52 |
| TPAB | | 0.012 | | 0.05 | | 0.56 |
| TBPN | | 0.012 | | 0.05 | | 0.56 |
| L5PN | | 0.009 | | -0.05 | | 0.57 |
| L5AB | | 0.009 | | -0.05 | | 0.57 |
| H4A1 | | 0.024 | | -0.05 | | 0.57 |
| V1CH | | 0.028 | | -0.05 | | 0.59 |
| TPTG | | 0.005 | | 0.05 | | 0.59 |
| HDTG | | 0.000 | | 0.04 | | 0.65 |
| V1TG | | 0.026 | | -0.03 | | 0.73 |
| LDHD | | 0.006 | | 0.03 | | 0.76 |
| L5FC | | 0.001 | | -0.03 | | 0.76 |
| L2TG | | 0.019 | | 0.02 | | 0.82 |
| IDAB | | 0.006 | | -0.02 | | 0.83 |
| IDPN | | 0.006 | | -0.02 | | 0.84 |
| V4PL | | 0.009 | | 0.01 | | 0.91 |
| H3TG | | 0.007 | | -0.01 | | 0.91 |
| VLFC | | 0.002 | | -0.01 | | 0.92 |

**Figure S2 – Multivariate model performance evaluation and classification accuracy of the OPLS-DA model for the non-burn control vs acute burn injury (Figure 1).** (A) Permutation test results showing the validation performance of the OPLS-DA model. The observed model parameters (R²Y and Q²Y) are compared with those obtained from randomly permuted class labels, demonstrating the robustness and statistical significance of the model. (B) Permutation test plot displaying the distribution of model performance after 999 random permutations. The low intercept values of R²Y and Q²Y (pR²Y = 0.001 and pQ² = 0.001) indicate that the original OPLS-DA model is unlikely to result from overfitting. (C) Observation diagnostics plot showing the score distance (SD) and orthogonal distance (OD) of individual samples. Samples within the defined confidence limits demonstrate acceptable model fit, while potential outliers are identified outside the threshold boundaries. (D) OPLS-DA scores plot showing separation between the Acute and recovered groups. The model explained 79.0% of the variation in X (R²X = 0.79), 68.2% of the variation in Y (R²Y = 0.682), and had a predictive ability of Q² = 0.619. (E) Receiver operating characteristic (ROC) and precision–recall (PR) curves evaluating the diagnostic performance of the classification model. The model achieved excellent discrimination ability with an area under the ROC curve (AUC-ROC) of 0.99 and an area under the precision–recall curve (AUC-PR) of 0.99, indicating high sensitivity, specificity, and predictive accuracy.

**Table S4 - OPLS loadings, Cliff’s delta and adjusted *p-*values of the lipoproteins for the comparison of the non-burn controls and healing patients.**

| **Lipoprotein** | **OPLS loadings** | | **Cliff’s delta** | | **Adjusted *p*-value** | |
| --- | --- | --- | --- | --- | --- | --- |
| H2A2 | | 0.189 | | -0.59 | | 2.35x10^-7^ |
| IDPL | | 0.185 | | -0.53 | | 2.75x10^-6^ |
| H3A2 | | 0.163 | | -0.52 | | 4.12x10^-6^ |
| H2PL | | 0.164 | | -0.51 | | 6.52x10^-6^ |
| H1A2 | | 0.156 | | -0.49 | | 1.43x10^-5^ |
| H1A1 | | 0.151 | | -0.48 | | 2.27x10^-5^ |
| H2CH | | 0.158 | | -0.48 | | 2.62x10^-5^ |
| H4FC | | 0.168 | | 0.46 | | 4.51x10^-5^ |
| L2AB | | 0.155 | | -0.45 | | 6.89x10^-5^ |
| L2PN | | 0.155 | | -0.45 | | 7.08x10^-5^ |
| L2FC | | 0.144 | | -0.45 | | 7.27x10^-5^ |
| L2CH | | 0.154 | | -0.44 | | 9.37x10^-5^ |
| V2CH | | 0.153 | | -0.44 | | 1.02x10^-4^ |
| L2PL | | 0.150 | | -0.44 | | 1.19x10^-4^ |
| H4A1 | | 0.146 | | 0.43 | | 1.30x10^-4^ |
| H4A2 | | 0.135 | | 0.42 | | 2.07x10^-4^ |
| H2FC | | 0.127 | | -0.42 | | 2.23x10^-4^ |
| H1FC | | 0.121 | | -0.40 | | 4.28x10^-4^ |
| H1PL | | 0.118 | | -0.38 | | 7.66x10^-4^ |
| L2TG | | 0.111 | | -0.38 | | 8.59x10^-4^ |
| H4CH | | 0.139 | | 0.37 | | 9.43x10^-4^ |
| HDPL | | 0.103 | | -0.37 | | 9.43x10^-4^ |
| V2PL | | 0.130 | | -0.37 | | 9.97x10^-4^ |
| H2A1 | | 0.104 | | -0.36 | | 1.35x10^-3^ |
| L6PN | | 0.081 | | 0.36 | | 1.66x10^-3^ |
| L6AB | | 0.081 | | 0.36 | | 1.66x10^-3^ |
| H4PL | | 0.126 | | 0.35 | | 2.07x10^-3^ |
| V1FC | | 0.128 | | -0.35 | | 2.25x10^-3^ |
| V2TG | | 0.119 | | -0.34 | | 2.68x10^-3^ |
| TPCH | | 0.111 | | -0.34 | | 2.73x10^-3^ |
| HDA1 | | 0.089 | | -0.32 | | 4.43x10^-3^ |
| L6CH | | 0.076 | | 0.31 | | 6.08x10^-3^ |
| H1CH | | 0.098 | | -0.31 | | 6.21x10^-3^ |
| HDCH | | 0.093 | | -0.31 | | 6.27x10^-3^ |
| V3PL | | 0.126 | | -0.31 | | 6.39x10^-3^ |
| H3PL | | 0.094 | | -0.31 | | 6.84x10^-3^ |
| IDPN | | 0.121 | | -0.30 | | 7.53x10^-3^ |
| L6TG | | 0.083 | | 0.30 | | 7.59x10^-3^ |
| IDAB | | 0.121 | | -0.30 | | 7.60x10^-3^ |
| L1FC | | 0.084 | | -0.30 | | 7.60x10^-2^ |
| VLPL | | 0.126 | | -0.30 | | 7.74x10^-2^ |
| L1CH | | 0.102 | | -0.30 | | 8.12x10^-2^ |
| L1PL | | 0.094 | | -0.29 | | 1.02x10^-2^ |
| HDFC | | 0.070 | | -0.29 | | 1.02x10^-2^ |
| L1TG | | 0.100 | | 0.28 | | 1.45x10^-2^ |
| L6FC | | 0.078 | | 0.26 | | 1.97x10^-2^ |
| L6PL | | 0.057 | | 0.26 | | 1.98x10^-2^ |
| V2FC | | 0.125 | | -0.26 | | 2.01x10^-2^ |
| H2TG | | 0.103 | | -0.26 | | 2.05x10^-2^ |
| V1PL | | 0.100 | | -0.26 | | 2.37x10^-2^ |
| TPA1 | | 0.064 | | -0.25 | | 2.66x10^-2^ |
| TPA2 | | 0.088 | | -0.25 | | 2.98x10^-2^ |
| L1AB | | 0.075 | | -0.25 | | 3.03x10^-2^ |
| L1PN | | 0.075 | | -0.25 | | 3.03x10^-2^ |
| H3CH | | 0.074 | | -0.24 | | 3.33x10^-2^ |
| IDFC | | 0.092 | | -0.24 | | 3.70x10^-2^ |
| V1CH | | 0.108 | | -0.24 | | 3.70x10^-2^ |
| HDA2 | | 0.084 | | -0.23 | | 3.97x10^-2^ |
| V4FC | | 0.095 | | -0.23 | | 4.11x10^-2^ |
| IDCH | | 0.094 | | -0.23 | | 4.48x10^-2^ |
| VLCH | | 0.108 | | -0.23 | | 4.49x10^-2^ |
| LDCH | | 0.082 | | -0.22 | | 5.53x10^-2^ |
| IDTG | | 0.104 | | -0.22 | | 5.61x10^-2^ |
| H3A1 | | 0.069 | | -0.20 | | 7.17x10^-2^ |
| V5FC | | 0.070 | | -0.20 | | 7.22x10^-2^ |
| H1TG | | 0.085 | | -0.20 | | 7.32x10^-2^ |
| H3TG | | 0.075 | | -0.20 | | 7.43x10^-2^ |
| TPTG | | 0.094 | | -0.19 | | 9.44x10^-2^ |
| V3FC | | 0.089 | | -0.17 | | 0.12 |
| LDFC | | 0.053 | | -0.17 | | 0.13 |
| LDPL | | 0.065 | | -0.17 | | 0.14 |
| LDTG | | 0.033 | | 0.16 | | 0.16 |
| V1TG | | 0.085 | | -0.16 | | 0.16 |
| V3CH | | 0.085 | | -0.16 | | 0.17 |
| VLFC | | 0.086 | | -0.15 | | 0.17 |
| V3TG | | 0.075 | | -0.15 | | 0.17 |
| VLTG | | 0.091 | | -0.15 | | 0.18 |
| L3AB | | 0.042 | | -0.15 | | 0.19 |
| L3PN | | 0.042 | | -0.15 | | 0.19 |
| L4FC | | 0.042 | | 0.15 | | 0.19 |
| VLAB | | 0.068 | | -0.14 | | 0.21 |
| L4PL | | 0.033 | | 0.14 | | 0.21 |
| VLPN | | 0.068 | | -0.14 | | 0.22 |
| L3CH | | 0.041 | | -0.13 | | 0.24 |
| L3PL | | 0.037 | | -0.13 | | 0.24 |
| H3FC | | 0.031 | | -0.13 | | 0.24 |
| ABA1 | | 0.003 | | 0.13 | | 0.25 |
| V4PL | | 0.067 | | -0.13 | | 0.26 |
| L3TG | | 0.034 | | -0.12 | | 0.29 |
| HDTG | | 0.058 | | -0.11 | | 0.33 |
| V5CH | | 0.025 | | 0.11 | | 0.35 |
| V4CH | | 0.056 | | -0.10 | | 0.38 |
| L4TG | | 0.006 | | 0.10 | | 0.38 |
| L4AB | | 0.017 | | 0.09 | | 0.43 |
| L4PN | | 0.017 | | 0.09 | | 0.42 |
| L4CH | | 0.016 | | 0.09 | | 0.43 |
| L5PL | | 0.051 | | -0.09 | | 0.43 |
| L3FC | | 0.008 | | -0.08 | | 0.46 |
| LDHD | | 0.015 | | 0.08 | | 0.46 |
| LDPN | | 0.036 | | -0.07 | | 0.51 |
| LDAB | | 0.036 | | -0.07 | | 0.51 |
| L5CH | | 0.047 | | -0.07 | | 0.53 |
| H4TG | | 0.019 | | 0.07 | | 0.53 |
| V5TG | | 0.022 | | -0.07 | | 0.56 |
| V5PL | | 0.024 | | -0.06 | | 0.57 |
| L5FC | | 0.010 | | 0.06 | | 0.59 |
| L5AB | | 0.038 | | -0.03 | | 0.78 |
| L5PN | | 0.038 | | -0.03 | | 0.78 |
| TPAB | | 0.029 | | -0.03 | | 0.82 |
| TBPN | | 0.029 | | -0.03 | | 0.82 |
| V4TG | | 0.024 | | -0.02 | | 0.83 |
| L5TG | | 0.038 | | 0.01 | | 0.94 |

**Table S5 - OPLS loadings, Cliff’s delta and adjusted *p*-values of the lipoproteins for the comparison of the non-burn controls and recovered patients.**

| **Lipoprotein** | **OPLS loadings** | | **Cliff’s delta** | | **Adjusted *p-*value** | |
| --- | --- | --- | --- | --- | --- | --- |
| H2CH | | 0.189 | | -0.47 | | 1.03x10^-5^ |
| V2CH | | 0.115 | | -0.46 | | 1.33x10^-5^ |
| L6TG | | 0.157 | | 0.46 | | 1.62x10^-5^ |
| H1A1 | | 0.171 | | -0.44 | | 4.19x10^-5^ |
| LDTG | | 0.131 | | 0.43 | | 5.66x10^-5^ |
| L6AB | | 0.153 | | 0.43 | | 6.47x10^-5^ |
| L6PN | | 0.153 | | 0.43 | | 6.47x10^-5^ |
| L2CH | | 0.180 | | -0.42 | | 7.60x10^-5^ |
| H2PL | | 0.180 | | -0.42 | | 8.51x10^-5^ |
| IDPL | | 0.140 | | -0.42 | | 8.89x10^-5^ |
| L2PL | | 0.174 | | -0.40 | | 1.59x10^-4^ |
| L2AB | | 0.176 | | -0.39 | | 2.49x10^-4^ |
| L2PN | | 0.176 | | -0.39 | | 2.52x10^-4^ |
| L2FC | | 0.166 | | -0.39 | | 2.60x10^-4^ |
| H2A2 | | 0.158 | | -0.39 | | 2.76x10^-4^ |
| H4FC | | 0.156 | | 0.38 | | 3.77x10^-4^ |
| HDCH | | 0.137 | | -0.38 | | 3.85x10^-4^ |
| L1TG | | 0.136 | | 0.38 | | 2.89x10^-4^ |
| L6CH | | 0.133 | | 0.37 | | 5.37x10^-4^ |
| V3PL | | 0.116 | | -0.37 | | 5.59x10^-4^ |
| V2PL | | 0.104 | | -0.36 | | 7.02x10^-4^ |
| L4FC | | 0.143 | | 0.36 | | 8.05x10^-4^ |
| H3A2 | | 0.121 | | -0.35 | | 1.04x10^-3^ |
| HDA1 | | 0.126 | | -0.35 | | 1.11x10^-3^ |
| H1A2 | | 0.140 | | -0.34 | | 1.56x10^-3^ |
| L1CH | | 0.125 | | -0.33 | | 1.78x10^-3^ |
| L4TG | | 0.097 | | 0.33 | | 2.26x10^-3^ |
| L6FC | | 0.122 | | 0.33 | | 2.31x10^-3^ |
| L6PL | | 0.125 | | 0.33 | | 2.31x10^-3^ |
| V1FC | | 0.092 | | -0.32 | | 2.81x10^-3^ |
| L1PL | | 0.115 | | -0.31 | | 3.84x10^-3^ |
| L4PL | | 0.123 | | 0.31 | | 4.08x10^-3^ |
| L4AB | | 0.116 | | 0.30 | | 4.46x10^-3^ |
| L4PN | | 0.116 | | 0.30 | | 4.54x10^-3^ |
| H1PL | | 0.125 | | -0.30 | | 4.96x10^-3^ |
| ABA1 | | 0.097 | | 0.30 | | 5.37x10^-3^ |
| V2TG | | 0.084 | | -0.29 | | 7.05x10^-3^ |
| VLPL | | 0.099 | | -0.29 | | 7.05x10^-3^ |
| HDPL | | 0.120 | | -0.28 | | 7.96x10^-3^ |
| H1CH | | 0.113 | | -0.27 | | 1.06x10^-2^ |
| H1FC | | 0.098 | | -0.27 | | 1.11x10^-2^ |
| L1FC | | 0.105 | | -0.27 | | 1.22x10^-2^ |
| TPA2 | | 0.090 | | -0.27 | | 1.23x10^-2^ |
| V5FC | | 0.085 | | -0.26 | | 1.26x10^-2^ |
| L5FC | | 0.101 | | 0.26 | | 1.45x10^-2^ |
| TPA1 | | 0.090 | | -0.25 | | 1.95x10^-2^ |
| HDA2 | | 0.083 | | -0.24 | | 2.43x10^-2^ |
| VLCH | | 0.077 | | -0.24 | | 2.62x10^-2^ |
| L4CH | | 0.102 | | 0.23 | | 2.79x10^-2^ |
| V3CH | | 0.062 | | -0.23 | | 2.87x10^-2^ |
| L1AB | | 0.085 | | -0.22 | | 3.53x10^-2^ |
| L1PN | | 0.085 | | -0.22 | | 3.53x10^-2^ |
| V3TG | | 0.063 | | -0.22 | | 3.63x10^-2^ |
| L5TG | | 0.058 | | 0.22 | | 3.63x10^-2^ |
| V4FC | | 0.056 | | -0.22 | | 3.74x10^-2^ |
| TPCH | | 0.085 | | -0.22 | | 3.90x10^-2^ |
| V4CH | | 0.057 | | -0.21 | | 4.49x10^-2^ |
| V3FC | | 0.057 | | -0.21 | | 4.58x10^-2^ |
| L2TG | | 0.098 | | -0.21 | | 4.64x10^-2^ |
| L3TG | | 0.068 | | 0.20 | | 6.29x10^-2^ |
| IDFC | | 0.038 | | -0.19 | | 7.17x10^-2^ |
| V4PL | | 0.046 | | -0.19 | | 7.52x10^-2^ |
| H2A1 | | 0.089 | | -0.19 | | 7.61x10^-2^ |
| LDHD | | 0.069 | | 0.18 | | 8.84x10^-2^ |
| IDTG | | 0.062 | | -0.17 | | 0.11 |
| IDPN | | 0.041 | | -0.16 | | 0.12 |
| IDAB | | 0.041 | | -0.16 | | 0.13 |
| H2FC | | 0.082 | | -0.16 | | 0.13 |
| LDCH | | 0.054 | | -0.16 | | 0.14 |
| TPAB | | 0.057 | | 0.16 | | 0.14 |
| TBPN | | 0.057 | | 0.16 | | 0.14 |
| H4A1 | | 0.084 | | 0.15 | | 0.17 |
| V5TG | | 0.043 | | 0.14 | | 0.18 |
| H3CH | | 0.058 | | -0.14 | | 0.18 |
| H3PL | | 0.078 | | -0.14 | | 0.18 |
| IDCH | | 0.024 | | -0.14 | | 0.19 |
| H4A2 | | 0.079 | | 0.14 | | 0.19 |
| V1PL | | 0.054 | | -0.14 | | 0.20 |
| H4PL | | 0.068 | | 0.13 | | 0.21 |
| LDPN | | 0.034 | | 0.13 | | 0.23 |
| LDAB | | 0.034 | | 0.13 | | 0.23 |
| L5AB | | 0.048 | | 0.13 | | 0.23 |
| H2TG | | 0.068 | | -0.13 | | 0.23 |
| L5PN | | 0.048 | | 0.13 | | 0.23 |
| VLFC | | 0.055 | | -0.13 | | 0.24 |
| VLPN | | 0.034 | | -0.12 | | 0.28 |
| VLAB | | 0.034 | | -0.12 | | 0.28 |
| H1TG | | 0.066 | | -0.11 | | 0.29 |
| HDFC | | 0.034 | | -0.11 | | 0.31 |
| V4TG | | 0.012 | | -0.09 | | 0.38 |
| VLTG | | 0.043 | | -0.09 | | 0.40 |
| V5CH | | 0.013 | | 0.09 | | 0.42 |
| V5FC | | 0.048 | | -0.08 | | 0.44 |
| LDPL | | 0.027 | | -0.08 | | 0.45 |
| V1CH | | 0.054 | | -0.07 | | 0.49 |
| TPTG | | 0.041 | | -0.07 | | 0.49 |
| L5CH | | 0.032 | | 0.07 | | 0.50 |
| H4CH | | 0.067 | | 0.07 | | 0.51 |
| L5PL | | 0.029 | | 0.06 | | 0.55 |
| H3A1 | | 0.037 | | -0.06 | | 0.56 |
| H3TG | | 0.039 | | -0.05 | | 0.65 |
| L3CH | | 0.017 | | -0.04 | | 0.68 |
| H3FC | | 0.016 | | -0.04 | | 0.70 |
| L3FC | | 0.017 | | 0.04 | | 0.73 |
| H4TG | | 0.003 | | -0.03 | | 0.75 |
| L3AB | | 0.003 | | 0.03 | | 0.80 |
| L3PN | | 0.003 | | 0.03 | | 0.80 |
| L3PL | | 0.010 | | -0.02 | | 0.86 |
| V1TG | | 0.029 | | 0.01 | | 0.90 |
| HDTG | | 0.032 | | 0.01 | | 0.91 |
| V5PL | | 0.019 | | -0.01 | | 0.92 |
| LDFC | | 0.002 | | 0.01 | | 0.95 |

**Figure S3 – Box plots of the main lipoprotein parameter concentrations and calculated ratios for the non-burn controls (blue), acute burn injury (red), healing (green) and recovered (green) burns patients.** (A) Total triglycerides (TPTG), (B) total cholesterol (TPCH), (C) low-density lipoprotein cholesterol (LDCH), (D) high-density lipoprotein cholesterol (HDCH), (E) total apolipoprotein A1 (TPA1), (F) total apolipoprotein A2 (TPA2), (G) total apolipoprotein B100 (TPAB), (H) LDL cholesterol/HDL cholesterol (LDHD), (I) Apolipoprotein B100/Apolipoprotein A1 (ABA1). Significant *p*-values are shown across groups (**p*<0.05; ***p*<0.01; ****p*<0.001; *****p*<0.0001) determined using Wilcoxon test.

**Table S6 – Group comparisons of total triglyceride (TPTG).**

| **Group comparisons** | **Adjusted *p-*value** |
| --- | --- |
| Control vs acute burn | 1.00 |
| Control vs healing | 0.11 |
| Control vs recovered | 0.04 |
| Acute burn vs healing | 0.01 |
| Acute burn vs recovered | 4.10x10^-4^ |
| Healing vs recovering | 1.00 |

**Table S7– Group comparisons of total cholesterol (TPCH).**

| **Group comparisons** | **Adjusted *p-*value** |
| --- | --- |
| Control vs acute burn | 3.60x10^-10^ |
| Control vs healing | 7.53x10^-4^ |
| Control vs recovered | 0.05 |
| Acute burn vs healing | 0.02 |
| Acute burn vs recovered | 5.40x10^-5^ |
| Healing vs recovering | 0.11 |

**Table S8 – Group comparisons of LDL cholesterol (LDCH).**

| **Group comparisons** | **Adjusted *p-*value** |
| --- | --- |
| Control vs acute burn | 3.00x10^-8^ |
| Control vs healing | 0.10 |
| Control vs recovered | 0.27 |
| Acute burn vs healing | 2.5x10^-3^ |
| Acute burn vs recovered | 9.70x10^-6^ |
| Healing vs recovering | 0.27 |

**Table S9 – Group comparisons of HDL cholesterol (HDCH).**

| **Group comparisons** | **Adjusted *p-*value** |
| --- | --- |
| Control vs acute burn | 8.80x10^-11^ |
| Control vs healing | 0.02 |
| Control vs recovered | 0.07 |
| Acute burn vs healing | 3.4x10^-4^ |
| Acute burn vs recovered | 1.80x10^-6^ |
| Healing vs recovering | 0.23 |

**Table S10 – Group comparisons of total apolipoprotein A1 (TPA1).**

| **Group comparisons** | **Adjusted *p-*value** |
| --- | --- |
| Control vs acute burn | 1.90x10^-10^ |
| Control vs healing | 0.04 |
| Control vs recovered | 0.30 |
| Acute burn vs healing | 2.4x10^-4^ |
| Acute burn vs recovered | 7.90x10^-7^ |
| Healing vs recovering | 0.30 |

**Table S11 – Group comparisons of total apolipoprotein A2 (TPA2).**

| **Group comparisons** | **Adjusted *p-*value** |
| --- | --- |
| Control vs acute burn | 7.60x10^-12^ |
| Control vs healing | 9.40x10^-3^ |
| Control vs recovered | 0.16 |
| Acute burn vs healing | 1.10x10^-4^ |
| Acute burn vs recovered | 1.00x10^-6^ |
| Healing vs recovering | 0.19 |

**Table S12 – Group comparisons of total apolipoprotein B100 (TPAB).**

| **Group comparisons** | **Adjusted *p-*value** |
| --- | --- |
| Control vs acute burn | 1.00 |
| Control vs healing | 1.00 |
| Control vs recovered | 1.00 |
| Acute burn vs healing | 1.00 |
| Acute burn vs recovered | 1.00 |
| Healing vs recovering | 1.00 |

**Table S13 – Group comparisons of LDL-cholesterol/HDL-cholesterol (LDHD).**

| **Group comparisons** | **Adjusted *p-*value** |
| --- | --- |
| Control vs acute burn | 1.00 |
| Control vs healing | 1.00 |
| Control vs recovered | 1.00 |
| Acute burn vs healing | 1.00 |
| Acute burn vs recovered | 1.00 |
| Healing vs recovering | 1.00 |

**Table S14 – Group comparisons of Apolipoprotein B100/Apolipoprotein A1 (ABA1).**

| **Group comparisons** | **Adjusted *p-*value** |
| --- | --- |
| Control vs acute burn | 1.60x10^-4^ |
| Control vs healing | 0.53 |
| Control vs recovered | 0.53 |
| Acute burn vs healing | 0.04 |
| Acute burn vs recovered | 0.04 |
| Healing vs recovering | 0.97 |

**Figure S4 – Box plots of the total concentration of Apolipoprotein B100 carrying particles for the non-burn controls (blue), acute burn injury (red), healing (green) and recovered (green) burns patients.** (A) Total particle number (TBPN), (B) very low-density lipoprotein particle number (VLPN), (C) intermediate density lipoprotein particle number (IDPN) (D) low density lipoprotein particle number (LDPN). Concentrations of VLDL, IDL and LDL particles are calculated from respective Apo-B100 concentrations considering that each VLDL, IDL and LDL particle carries one apolipoprotein B100 molecule only. (**p*<0.05; ***p*<0.01; ****p*<0.001; *****p*<0.0001) determined using Wilcoxon test.

**Table S15 – Group comparisons of total particle number (TBPN).**

| **Group comparisons** | **Adjusted *p-*value** |
| --- | --- |
| Control vs acute burn | 1.00 |
| Control vs healing | 1.00 |
| Control vs recovered | 1.00 |
| Acute burn vs healing | 1.00 |
| Acute burn vs recovered | 1.00 |
| Healing vs recovering | 1.00 |

**Table S16 – Group comparisons of very low-density particle number (VLPN).**

| **Group comparisons** | **Adjusted *p-*value** |
| --- | --- |
| Control vs acute burn | 0.15 |
| Control vs healing | 0.15 |
| Control vs recovered | 7.50x10^-3^ |
| Acute burn vs healing | 2.50x10^-4^ |
| Acute burn vs recovered | 4.70x10^-7^ |
| Healing vs recovering | 0.15 |

**Table S17 – Group comparisons of intermediate density particle number (IDPN).**

| **Group comparisons** | **Adjusted *p-*value** |
| --- | --- |
| Control vs acute burn | 1.00 |
| Control vs healing | 4.30x10^-3^ |
| Control vs recovered | 4.30x10^-3^ |
| Acute burn vs healing | 4.30x10^-3^ |
| Acute burn vs recovered | 5.20x10^-3^ |
| Healing vs recovering | 1.00 |

**Table S18 – Group comparisons of low-density particle number (LDPN).**

| **Group comparisons** | **Adjusted *p-*value** |
| --- | --- |
| Control vs acute burn | 0.64 |
| Control vs healing | 1.00 |
| Control vs recovered | 1.00 |
| Acute burn vs healing | 1.00 |
| Acute burn vs recovered | 0.39 |
| Healing vs recovering | 0.95 |

**Figure S5 – Box plots of LDL particle number for the non-burn controls (blue), acute burn injury (red), healing (green) and recovered (green) burns patients.** (A) LDL-1 particle number (L1PN), (B) LDL-2 particle number (L2PN), (C) LDL-3 particle number (L3PN), (D) LDL-4 particle number (L4PN), (E) LDL-5 particle number (L5PN), (F) LDL-6 particle number (L6PN). (**p*<0.05; ***p*<0.01; ****p*<0.001; *****p*<0.0001) determined using Wilcoxon test.

**Table S19 – Group comparisons of LDL subfraction 1 particle number (L1PN).**

| **Group comparisons** | **Adjusted *p-*value** |
| --- | --- |
| Control vs acute burn | 0.77 |
| Control vs healing | 0.23 |
| Control vs recovered | 0.41 |
| Acute burn vs healing | 0.77 |
| Acute burn vs recovered | 0.77 |
| Healing vs recovering | 0.77 |

**Table S20 – Group comparisons of LDL subfraction 2 particle number (L2PN).**

| **Group comparisons** | **Adjusted *p-*value** |
| --- | --- |
| Control vs acute burn | 5.50x10^-9^ |
| Control vs healing | 2.60x10^-3^ |
| Control vs recovered | 0.01 |
| Acute burn vs healing | 1.50x10^-3^ |
| Acute burn vs recovered | 4.50x10^-5^ |
| Healing vs recovering | 0.23 |

**Table S21 – Group comparisons of LDL subfraction 3 particle number (L3PN).**

| **Group comparisons** | **Adjusted *p-*value** |
| --- | --- |
| Control vs acute burn | 8.80x10^-4^ |
| Control vs healing | 0.36 |
| Control vs recovered | 0.51 |
| Acute burn vs healing | 0.12 |
| Acute burn vs recovered | 4.10x10^-5^ |
| Healing vs recovering | 0.12 |

**Table S22 – Group comparisons of LDL subfraction 4 particle number (L4PN).**

| **Group comparisons** | **Adjusted *p-*value** |
| --- | --- |
| Control vs acute burn | 0.85 |
| Control vs healing | 0.89 |
| Control vs recovered | 0.86 |
| Acute burn vs healing | 0.86 |
| Acute burn vs recovered | 0.09 |
| Healing vs recovering | 0.86 |

**Table S23 – Group comparisons of LDL subfraction 5 particle number (L5PN).**

| **Group comparisons** | **Adjusted *p-*value** |
| --- | --- |
| Control vs acute burn | 1.00 |
| Control vs healing | 1.00 |
| Control vs recovered | 1.00 |
| Acute burn vs healing | 1.00 |
| Acute burn vs recovered | 1.00 |
| Healing vs recovering | 1.00 |

**Table S24 – Group comparisons of LDL subfraction 6 particle number (L6PN).**

| **Group comparisons** | **Adjusted *p-*value** |
| --- | --- |
| Control vs acute burn | 1.30x10^-4^ |
| Control vs healing | 1.10x10^-4^ |
| Control vs recovered | 6.00x10^-4^ |
| Acute burn vs healing | 1.00 |
| Acute burn vs recovered | 1.00 |
| Healing vs recovering | 1.00 |

**Figure S6 – Box plots of the total concentration of triglycerides in the main lipoprotein fractions for the non-burn controls (blue), acute burn injury (red), healing (green) and recovered (green) burns patients.** (A) VLDL triglyceride (VLTG), (B) IDL triglyceride (IDTG), (C) LDL triglyceride (LDTG), (D) HDL triglyceride (HDTG). (**p*<0.05; ***p*<0.01; ****p*<0.001; *****p*<0.0001) determined using Wilcoxon test.

**Table S25 – Group comparisons of VLDL triglyceride (VLTG).**

| **Group comparisons** | **Adjusted *p-*value** |
| --- | --- |
| Control vs acute burn | 0.70 |
| Control vs healing | 0.25 |
| Control vs recovered | 0.04 |
| Acute burn vs healing | 0.25 |
| Acute burn vs recovered | 0.02 |
| Healing vs recovering | 0.70 |

**Table S26 – Group comparisons of IDL triglyceride (IDTG).**

| **Group comparisons** | **Adjusted *p-*value** |
| --- | --- |
| Control vs acute burn | 0.42 |
| Control vs healing | 0.06 |
| Control vs recovered | 0.02 |
| Acute burn vs healing | 0.21 |
| Acute burn vs recovered | 0.08 |
| Healing vs recovering | 0.77 |

**Table S27 – Group comparisons of LDL triglyceride (LDTG).**

| **Group comparisons** | **Adjusted *p-*value** |
| --- | --- |
| Control vs acute burn | 1.50x10^-9^ |
| Control vs healing | 0.21 |
| Control vs recovered | 0.10 |
| Acute burn vs healing | 1.10x10^-6^ |
| Acute burn vs recovered | 2.50x10^-5^ |
| Healing vs recovering | 0.49 |

**Table S28 – Group comparisons of HDL triglyceride (HDTG).**

| **Group comparisons** | **Adjusted *p-*value** |
| --- | --- |
| Control vs acute burn | 0.98 |
| Control vs healing | 0.68 |
| Control vs recovered | 0.98 |
| Acute burn vs healing | 0.11 |
| Acute burn vs recovered | 0.25 |
| Healing vs recovering | 0.98 |

**Figure S7 – Box plots of the total concentration of cholesterol in the main lipoprotein fractions for the non-burn controls (blue), acute burn injury (red), healing (green) and recovered (green) burns patients.** (A) VLDL cholesterol (VLCH), (B) IDL cholesterol (IDCH), (C) LDL cholesterol (LDCH), (D) HDL cholesterol (HDCH). (**p*<0.05; ***p*<0.01; ****p*<0.001; *****p*<0.0001) determined using Wilcoxon test.

**Table S29 – Group comparisons of VLDL cholesterol (VLCH).**

| **Group comparisons** | **Adjusted *p-*value** |
| --- | --- |
| Control vs acute burn | 0.49 |
| Control vs healing | 0.02 |
| Control vs recovered | 3.00x10^-4^ |
| Acute burn vs healing | 0.03 |
| Acute burn vs recovered | 5.60x10^-5^ |
| Healing vs recovering | 0.30 |

**Table S30 – Group comparisons of IDL cholesterol (IDCH).**

| **Group comparisons** | **Adjusted *p-*value** |
| --- | --- |
| Control vs acute burn | 0.43 |
| Control vs healing | 0.04 |
| Control vs recovered | 0.02 |
| Acute burn vs healing | 0.43 |
| Acute burn vs recovered | 0.27 |
| Healing vs recovering | 0.65 |

**Table S31 – Group comparisons of LDL cholesterol (LDCH).**

| **Group comparisons** | **Adjusted *p-*value** |
| --- | --- |
| Control vs acute burn | 3.00x10^-8^ |
| Control vs healing | 0.10 |
| Control vs recovered | 0.27 |
| Acute burn vs healing | 2.50x10^-3^ |
| Acute burn vs recovered | 9.70x10^-6^ |
| Healing vs recovering | 0.27 |

**Table S32 – Group comparisons of HDL cholesterol (HDCH).**

| **Group comparisons** | **Adjusted *p-*value** |
| --- | --- |
| Control vs acute burn | 8.80x10^-11^ |
| Control vs healing | 0.02 |
| Control vs recovered | 0.07 |
| Acute burn vs healing | 3.40x10^-4^ |
| Acute burn vs recovered | 1.80x10^-6^ |
| Healing vs recovering | 0.23 |

**Figure S8 – Box plots of the total concentration of free cholesterol in the main lipoprotein fractions for the non-burn controls (blue), acute burn injury (red), healing (green) and recovered (green) burns patients.** (A) VLDL free cholesterol (VLFC), (B) IDL cholesterol (IDFC), (C) LDL cholesterol (LDFC), (D) HDL cholesterol (HDFC). (**p*<0.05; ***p*<0.01; ****p*<0.001; *****p*<0.0001) determined using Wilcoxon test.

**Table S33 – Group comparisons of VLDL free cholesterol (VLFC).**

| **Group comparisons** | **Adjusted *p-*value** |
| --- | --- |
| Control vs acute burn | 0.88 |
| Control vs healing | 0.13 |
| Control vs recovered | 8.40x10^-3^ |
| Acute burn vs healing | 0.06 |
| Acute burn vs recovered | 2.40x10^-4^ |
| Healing vs recovering | 0.23 |

**Table S34 – Group comparisons of IDL free cholesterol (IDFC).**

| **Group comparisons** | **Adjusted *p-*value** |
| --- | --- |
| Control vs acute burn | 0.14 |
| Control vs healing | 0.20 |
| Control vs recovered | 8.10x10^-3^ |
| Acute burn vs healing | 0.45 |
| Acute burn vs recovered | 0.28 |
| Healing vs recovering | 0.69 |

**Table S35 – Group comparisons of LDL free cholesterol (LDFC).**

| **Group comparisons** | **Adjusted *p-*value** |
| --- | --- |
| Control vs acute burn | 8.10x10^-5^ |
| Control vs healing | 0.34 |
| Control vs recovered | 0.80 |
| Acute burn vs healing | 0.02 |
| Acute burn vs recovered | 1.20x10^-4^ |
| Healing vs recovering | 0.34 |

**Table S36 – Group comparisons of HDL free cholesterol (HDFC).**

| **Group comparisons** | **Adjusted *p-*value** |
| --- | --- |
| Control vs acute burn | 4.10x10^-5^ |
| Control vs healing | 0.15 |
| Control vs recovered | 0.83 |
| Acute burn vs healing | 0.22 |
| Acute burn vs recovered | 5.50x10^-4^ |
| Healing vs recovering | 0.22 |

**Figure S9 – Box plots of the total concentration of phospholipid in the main lipoprotein fractions for the non-burn controls (blue), acute burn injury (red), healing (green) and recovered (green) burns patients.** (A) VLDL phospholipid (VLPL), (B) IDL phospholipid (IDPL), (C) LDL phospholipid (LDPL), (D) HDL phospholipid (HDPL). (**p*<0.05; ***p*<0.01; ****p*<0.001; *****p*<0.0001) determined using Wilcoxon text.

**Table S37– Group comparisons of VLDL phospholipid (VLPL).**

| **Group comparisons** | **Adjusted *p-*value** |
| --- | --- |
| Control vs acute burn | 0.08 |
| Control vs healing | 1.70x10^-3^ |
| Control vs recovered | 2.00x10^-5^ |
| Acute burn vs healing | 0.08 |
| Acute burn vs recovered | 1.70x10^-3^ |
| Healing vs recovering | 0.18 |

**Table S38 – Group comparisons of IDL phospholipid (IDPL).**

| **Group comparisons** | **Adjusted *p-*value** |
| --- | --- |
| Control vs acute burn | 8.80x10^-13^ |
| Control vs healing | 5.20x10^-7^ |
| Control vs recovered | 9.00x10^-7^ |
| Acute burn vs healing | 0.28 |
| Acute burn vs recovered | 0.24 |
| Healing vs recovering | 0.78 |

**Table S39 – Group comparisons of LDL phospholipid (LDPL).**

| **Group comparisons** | **Adjusted *p-*value** |
| --- | --- |
| Control vs acute burn | 1.40x10^-5^ |
| Control vs healing | 0.18 |
| Control vs recovered | 0.47 |
| Acute burn vs healing | 0.04 |
| Acute burn vs recovered | 2.40x10^-4^ |
| Healing vs recovering | 0.47 |

**Table S40 – Group comparisons of HDL phospholipid (HDPL).**

| **Group comparisons** | **Adjusted *p-*value** |
| --- | --- |
| Control vs acute burn | 4.30x10^-10^ |
| Control vs healing | 1.00x10^-3^ |
| Control vs recovered | 0.07 |
| Acute burn vs healing | 0.05 |
| Acute burn vs recovered | 4.50x10^-6^ |
| Healing vs recovering | 0.05 |

**Figure S10 – Box plots of the total concentration of Apolipoprotein A1 and Apolipoprotein B100 in the main lipoprotein fractions for the non-burn controls (blue), acute burn injury (red), healing (green) and recovered (green) burns patients.** (A) HDL Apo-A1 (HDA1), (B) HDL Apo-A2 (HDA2), (C) VLDL Apo-B100 (VLAB), (D) IDL Apo-B100 (IDAB), (E) LDL Apo-B100 (LDAB). (**p*<0.05; ***p*<0.01; ****p*<0.001; *****p*<0.0001) determined using Wilcoxon test.

**Table S41 – Group comparisons of HDL Apo-A1 (HDA1).**

| **Group comparisons** | **Adjusted *p-*value** |
| --- | --- |
| Control vs acute burn | 3.50x10^-12^ |
| Control vs healing | 6.50x10^-3^ |
| Control vs recovered | 0.06 |
| Acute burn vs healing | 7.90x10^-5^ |
| Acute burn vs recovered | 3.90x10^-7^ |
| Healing vs recovering | 0.20 |

**Table S42 – Group comparisons of HDL Apo-A2 (HDA2).**

| **Group comparisons** | **Adjusted *p-*value** |
| --- | --- |
| Control vs acute burn | 1.40x10^-10^ |
| Control vs healing | 9.70x10^-3^ |
| Control vs recovered | 0.23 |
| Acute burn vs healing | 5.20x10^-4^ |
| Acute burn vs recovered | 7.20x10^-6^ |
| Healing vs recovering | 0.23 |

**Table S43 – Group comparisons of VLDL Apo-B100 (VLAB).**

| **Group comparisons** | **Adjusted *p-*value** |
| --- | --- |
| Control vs acute burn | 0.15 |
| Control vs healing | 0.15 |
| Control vs recovered | 7.50x10^-3^ |
| Acute burn vs healing | 2.40x10^-4^ |
| Acute burn vs recovered | 4.60x10^-7^ |
| Healing vs recovering | 0.15 |

**Table S44 – Group comparisons of IDL Apo-B100 (IDAB).**

| **Group comparisons** | **Adjusted *p-*value** |
| --- | --- |
| Control vs acute burn | 1.00 |
| Control vs healing | 4.10x10^-3^ |
| Control vs recovered | 4.10x10^-3^ |
| Acute burn vs healing | 4.10x10^-3^ |
| Acute burn vs recovered | 5.50x10^-3^ |
| Healing vs recovering | 1.00 |

**Table S45 – Group comparisons of LDL Apo-B100 (LDAB).**

| **Group comparisons** | **Adjusted *p-*value** |
| --- | --- |
| Control vs acute burn | 0.64 |
| Control vs healing | 1.00 |
| Control vs recovered | 1.00 |
| Acute burn vs healing | 1.00 |
| Acute burn vs recovered | 0.39 |
| Healing vs recovering | 0.95 |

**Figure S11 – Box plots of the triglyceride concentration in the VLDL subfractions for the non-burn controls (blue), acute burn injury (red), healing (green) and recovered (green) burns patients.** (A) VLDL subfraction 1 triglyceride (V1TG), (B) VLDL subfraction 2 triglyceride (V2TG) (C) VLDL subfraction 3 triglyceride (V3TG), (D) VLDL subfraction 4 triglyceride (V4TG), (E) VLDL subfraction 5 triglyceride (V5TG). (**p*<0.05; ***p*<0.01; ****p*<0.001; *****p*<0.0001) determined using Wilcoxon test.

**Table S46 – Group comparisons of VLDL subfraction 1 triglyceride (V1TG).**

| **Group comparisons** | **Adjusted *p-*value** |
| --- | --- |
| Control vs acute burn | 1.00 |
| Control vs healing | 0.42 |
| Control vs recovered | 0.42 |
| Acute burn vs healing | 0.37 |
| Acute burn vs recovered | 0.37 |
| Healing vs recovering | 1.00 |

**Table S47 – Group comparisons of VLDL subfraction 2 triglyceride (V2TG).**

| **Group comparisons** | **Adjusted *p-*value** |
| --- | --- |
| Control vs acute burn | 0.04 |
| Control vs healing | 5.50x10^-3^ |
| Control vs recovered | 2.90x10^-4^ |
| Acute burn vs healing | 0.33 |
| Acute burn vs recovered | 0.06 |
| Healing vs recovering | 0.42 |

**Table S48 – Group comparisons of VLDL subfraction 3 triglyceride (V3TG).**

| **Group comparisons** | **Adjusted *p-*value** |
| --- | --- |
| Control vs acute burn | 0.44 |
| Control vs healing | 0.19 |
| Control vs recovered | 3.50x10^-3^ |
| Acute burn vs healing | 0.32 |
| Acute burn vs recovered | 7.50x10^-3^ |
| Healing vs recovering | 0.32 |

**Table S49 – Group comparisons of VLDL subfraction 4 triglyceride (V4TG).**

| **Group comparisons** | **Adjusted *p-*value** |
| --- | --- |
| Control vs acute burn | 0.41 |
| Control vs healing | 0.41 |
| Control vs recovered | 0.02 |
| Acute burn vs healing | 0.12 |
| Acute burn vs recovered | 1.80x10^-4^ |
| Healing vs recovering | 0.19 |

**Table S50 – Group comparisons of VLDL subfraction 5 triglyceride (V5TG).**

| **Group comparisons** | **Adjusted *p-*value** |
| --- | --- |
| Control vs acute burn | 1.60x10^-4^ |
| Control vs healing | 1.00 |
| Control vs recovered | 1.00 |
| Acute burn vs healing | 1.40x10^-5^ |
| Acute burn vs recovered | 1.60x10^-5^ |
| Healing vs recovering | 1.00 |

**Figure S12 – Box plots of the cholesterol concentration in the VLDL subfractions for the non-burn controls (blue), acute burn injury (red), healing (green) and recovered (green) burns patients.** (A) VLDL subfraction 1 cholesterol (V1CH), (B) VLDL subfraction 2 cholesterol (V2CH) (C) VLDL subfraction 3 cholesterol (V3CH), (D) VLDL subfraction 4 cholesterol (V4CH), (E) VLDL subfraction 5 cholesterol (V5CH). (**p*<0.05; ***p*<0.01; ****p*<0.001; *****p*<0.0001) determined using Wilcoxon test.

**Table S51 – Group comparisons of VLDL subfraction 1 cholesterol (V1CH).**

| **Group comparisons** | **Adjusted *p-*value** |
| --- | --- |
| Control vs acute burn | 1.00 |
| Control vs healing | 0.04 |
| Control vs recovered | 0.04 |
| Acute burn vs healing | 0.04 |
| Acute burn vs recovered | 2.90x10^-3^ |
| Healing vs recovering | 1.00 |

**Table S52 – Group comparisons of VLDL subfraction 2 cholesterol (V2CH).**

| **Group comparisons** | **Adjusted *p-*value** |
| --- | --- |
| Control vs acute burn | 6.30x10^-3^ |
| Control vs healing | 2.60x10^-5^ |
| Control vs recovered | 5.80x10^-8^ |
| Acute burn vs healing | 0.05 |
| Acute burn vs recovered | 1.10x10^-3^ |
| Healing vs recovering | 0.28 |

**Table S53 – Group comparisons of VLDL subfraction 3 cholesterol (V3CH).**

| **Group comparisons** | **Adjusted *p-*value** |
| --- | --- |
| Control vs acute burn | 0.41 |
| Control vs healing | 0.10 |
| Control vs recovered | 1.40x10^-3^ |
| Acute burn vs healing | 0.29 |
| Acute burn vs recovered | 9.60x10^-3^ |
| Healing vs recovering | 0.41 |

**Table S54 – Group comparisons of VLDL subfraction 4 cholesterol (V4CH).**

| **Group comparisons** | **Adjusted *p-*value** |
| --- | --- |
| Control vs acute burn | 0.51 |
| Control vs healing | 0.19 |
| Control vs recovered | 4.50x10^-4^ |
| Acute burn vs healing | 0.09 |
| Acute burn vs recovered | 3.00x10^-5^ |
| Healing vs recovering | 0.06 |

**Table S55 – Group comparisons of VLDL subfraction 5 cholesterol (V5CH).**

| **Group comparisons** | **Adjusted *p-*value** |
| --- | --- |
| Control vs acute burn | 9.50x10^-3^ |
| Control vs healing | 0.88 |
| Control vs recovered | 0.88 |
| Acute burn vs healing | 0.03 |
| Acute burn vs recovered | 2.90x10^-4^ |
| Healing vs recovering | 0.88 |

**Figure S13 – Box plots of the free cholesterol concentration in the VLDL subfractions for the non-burn controls (blue), acute burn injury (red), healing (green) and recovered (green) burns patients.** (A) VLDL subfraction 1 free cholesterol (V1FC), (B) VLDL subfraction 2 free cholesterol (V2FC) (C) VLDL subfraction 3 free cholesterol (V3FC), (D) VLDL subfraction 4 free cholesterol (V4FC), (E) VLDL subfraction 5 free cholesterol (V5FC). (**p*<0.05; ***p*<0.01; ****p*<0.001; *****p*<0.0001) determined using Wilcoxon test.

**Table S56 – Group comparisons of VLDL subfraction 1 free cholesterol (V1FC).**

| **Group comparisons** | **Adjusted *p-*value** |
| --- | --- |
| Control vs acute burn | 1.10x10^-4^ |
| Control vs healing | 1.50x10^-3^ |
| Control vs recovered | 8.90x10^-5^ |
| Acute burn vs healing | 1.00 |
| Acute burn vs recovered | 1.00 |
| Healing vs recovering | 1.00 |

**Table S57 – Group comparisons of VLDL subfraction 2 free cholesterol (V2FC).**

| **Group comparisons** | **Adjusted *p-*value** |
| --- | --- |
| Control vs acute burn | 0.53 |
| Control vs healing | 0.02 |
| Control vs recovered | 2.70x10^-4^ |
| Acute burn vs healing | 0.02 |
| Acute burn vs recovered | 1.90x10^-4^ |
| Healing vs recovering | 0.53 |

**Table S58 – Group comparisons of VLDL subfraction 3 free cholesterol (V3FC).**

| **Group comparisons** | **Adjusted *p-*value** |
| --- | --- |
| Control vs acute burn | 0.50 |
| Control vs healing | 0.12 |
| Control vs recovered | 1.90x10^-3^ |
| Acute burn vs healing | 0.12 |
| Acute burn vs recovered | 6.40x10^-4^ |
| Healing vs recovering | 0.39 |

**Table S59 – Group comparisons of VLDL subfraction 4 free cholesterol (V4FC).**

| **Group comparisons** | **Adjusted *p-*value** |
| --- | --- |
| Control vs acute burn | 0.38 |
| Control vs healing | 0.02 |
| Control vs recovered | 3.60x10^-4^ |
| Acute burn vs healing | 0.11 |
| Acute burn vs recovered | 1.90x10^-3^ |
| Healing vs recovering | 0.38 |

**Table S60 – Group comparisons of VLDL subfraction 5 free cholesterol (V5FC).**

| **Group comparisons** | **Adjusted *p-*value** |
| --- | --- |
| Control vs acute burn | 0.20 |
| Control vs healing | 0.08 |
| Control vs recovered | 0.04 |
| Acute burn vs healing | 1.10x10^-3^ |
| Acute burn vs recovered | 1.00x10^-4^ |
| Healing vs recovering | 0.97 |

**Figure S14 – Box plots of the phospholipid concentration in the VLDL subfractions for the non-burn controls (blue), acute burn injury (red), healing (green) and recovered (green) burns patients.** (A) VLDL subfraction 1 phospholipid (V1PL), (B) VLDL subfraction 2 phospholipid (V2PL) (C) VLDL subfraction 3 phospholipid (V3PL), (D) VLDL subfraction 4 phospholipid (V4PL), (E) VLDL subfraction 5 phospholipid (V5PL). (**p*<0.05; ***p*<0.01; ****p*<0.001; *****p*<0.0001) determined using Wilcoxon test.

**Table S61 – Group comparisons of VLDL subfraction 1 phospholipid (V1PL).**

| **Group comparisons** | **Adjusted *p-*value** |
| --- | --- |
| Control vs acute burn | 0.20 |
| Control vs healing | 0.02 |
| Control vs recovered | 0.01 |
| Acute burn vs healing | 0.19 |
| Acute burn vs recovered | 0.09 |
| Healing vs recovering | 0.79 |

**Table S62 – Group comparisons of VLDL subfraction 2 phospholipid (V2PL).**

| **Group comparisons** | **Adjusted *p-*value** |
| --- | --- |
| Control vs acute burn | 1.10x10^-3^ |
| Control vs healing | 5.30x10^-4^ |
| Control vs recovered | 1.60x10^-5^ |
| Acute burn vs healing | 0.50 |
| Acute burn vs recovered | 0.08 |
| Healing vs recovering | 0.50 |

**Table S63 – Group comparisons of VLDL subfraction 3 phospholipid (V3PL).**

| **Group comparisons** | **Adjusted *p-*value** |
| --- | --- |
| Control vs acute burn | 2.60x10^-3^ |
| Control vs healing | 1.50x10^-3^ |
| Control vs recovered | 6.00x10^-6^ |
| Acute burn vs healing | 0.35 |
| Acute burn vs recovered | 0.02 |
| Healing vs recovering | 0.21 |

**Table S64 – Group comparisons of VLDL subfraction 4 phospholipid (V4PL).**

| **Group comparisons** | **Adjusted *p-*value** |
| --- | --- |
| Control vs acute burn | 0.72 |
| Control vs healing | 0.13 |
| Control vs recovered | 7.40x10^-4^ |
| Acute burn vs healing | 0.04 |
| Acute burn vs recovered | 4.50x10^-5^ |
| Healing vs recovering | 0.13 |

**Table S65 – Group comparisons of VLDL subfraction 5 phospholipid (V5PL).**

| **Group comparisons** | **Adjusted *p-*value** |
| --- | --- |
| Control vs acute burn | 0.27 |
| Control vs healing | 0.37 |
| Control vs recovered | 0.12 |
| Acute burn vs healing | 0.02 |
| Acute burn vs recovered | 2.00x10^-4^ |
| Healing vs recovering | 0.37 |

**Figure S15 – Box plots of the triglyceride concentration in the LDL subfractions for the non-burn controls (blue), acute burn injury (red), healing (green) and recovered (green) burns patients.** (A) LDL subfraction 1 triglyceride (L1TG), (B) LDL subfraction 2 triglyceride (L2TG), (C) LDL subfraction 3 triglyceride (L3TG), (D) LDL subfraction 4 triglyceride (L4TG), (E) LDL subfraction 5 triglyceride (L5TG), (F) LDL subfraction 6 triglyceride (L6TG). (**p*<0.05; ***p*<0.01; ****p*<0.001; *****p*<0.0001) determined using Wilcoxon test.

**Table S66 – Group comparisons of LDL subfraction 1 triglyceride (L1TG).**

| **Group comparisons** | **Adjusted *p-*value** |
| --- | --- |
| Control vs acute burn | 4.50x10^-13^ |
| Control vs healing | 0.03 |
| Control vs recovered | 0.03 |
| Acute burn vs healing | 4.80x10^-6^ |
| Acute burn vs recovered | 1.50x10^-5^ |
| Healing vs recovering | 0.88 |

**Table S67 – Group comparisons of LDL subfraction 2 triglyceride (L2TG).**

| **Group comparisons** | **Adjusted *p-*value** |
| --- | --- |
| Control vs acute burn | 0.93 |
| Control vs healing | 4.70x10^-3^ |
| Control vs recovered | 0.02 |
| Acute burn vs healing | 4.20x10^-3^ |
| Acute burn vs recovered | 1.90x10^-2^ |
| Healing vs recovering | 0.93 |

**Table S68 – Group comparisons of LDL subfraction 3 triglyceride (L3TG).**

| **Group comparisons** | **Adjusted *p-*value** |
| --- | --- |
| Control vs acute burn | 1.80x10^-5^ |
| Control vs healing | 0.87 |
| Control vs recovered | 0.87 |
| Acute burn vs healing | 2.90x10^-6^ |
| Acute burn vs recovered | 4.50x10^-4^ |
| Healing vs recovering | 0.71 |

**Table S69 – Group comparisons of LDL subfraction 4 triglyceride (L4TG).**

| **Group comparisons** | **Adjusted *p-*value** |
| --- | --- |
| Control vs acute burn | 1.50x10^-3^ |
| Control vs healing | 1.00 |
| Control vs recovered | 1.00 |
| Acute burn vs healing | 1.50x10^-3^ |
| Acute burn vs recovered | 7.80x10^-3^ |
| Healing vs recovering | 1.00 |

**Table S70 – Group comparisons of LDL subfraction 5 triglyceride (L5TG).**

| **Group comparisons** | **Adjusted *p-*value** |
| --- | --- |
| Control vs acute burn | 6.40x10^-3^ |
| Control vs healing | 1.00 |
| Control vs recovered | 1.00 |
| Acute burn vs healing | 1.60x10^-4^ |
| Acute burn vs recovered | 1.60x10^-4^ |
| Healing vs recovering | 1.00 |

**Table S71 – Group comparisons of LDL subfraction 6 triglyceride (L6TG).**

| **Group comparisons** | **Adjusted *p-*value** |
| --- | --- |
| Control vs acute burn | 6.50x10^-11^ |
| Control vs healing | 4.20x10^-4^ |
| Control vs recovered | 6.60x10^-4^ |
| Acute burn vs healing | 0.02 |
| Acute burn vs recovered | 0.02 |
| Healing vs recovering | 0.77 |

**Figure S16 – Box plots of the cholesterol concentration in the LDL subfractions for the non-burn controls (blue), acute burn injury (red), healing (green) and recovered (green) burns patients.** (A) LDL subfraction 1 cholesterol (L1CH), (B) LDL subfraction 2 cholesterol (L2CH), (C) LDL subfraction 3 cholesterol (L3CH), (D) LDL subfraction 4 cholesterol (L4CH), (E) LDL subfraction 5 cholesterol (L5CH), (F) LDL subfraction 6 cholesterol (L6CH). (**p*<0.05; ***p*<0.01; ****p*<0.001; *****p*<0.0001) determined using Wilcoxon test.

**Table S72 – Group comparisons of LDL subfraction 1 cholesterol (L1CH).**

| **Group comparisons** | **Adjusted *p-*value** |
| --- | --- |
| Control vs acute burn | 9.80x10^-5^ |
| Control vs healing | 0.03 |
| Control vs recovered | 0.04 |
| Acute burn vs healing | 0.68 |
| Acute burn vs recovered | 0.31 |
| Healing vs recovering | 0.68 |

**Table S73 – Group comparisons of LDL subfraction 2 cholesterol (L2CH).**

| **Group comparisons** | **Adjusted *p-*value** |
| --- | --- |
| Control vs acute burn | 3.20x10^-11^ |
| Control vs healing | 2.80x10^-3^ |
| Control vs recovered | 9.30x10^-3^ |
| Acute burn vs healing | 8.30x10^-6^ |
| Acute burn vs recovered | 2.00x10^-7^ |
| Healing vs recovering | 0.38 |

**Table S74 – Group comparisons of LDL subfraction 3 cholesterol (L3CH).**

| **Group comparisons** | **Adjusted *p-*value** |
| --- | --- |
| Control vs acute burn | 2.10x10^-6^ |
| Control vs healing | 0.41 |
| Control vs recovered | 0.65 |
| Acute burn vs healing | 1.90x10^-3^ |
| Acute burn vs recovered | 1.00x10^-7^ |
| Healing vs recovering | 0.24 |

**Table S75 – Group comparisons of LDL subfraction 4 cholesterol (L4CH).**

| **Group comparisons** | **Adjusted *p-*value** |
| --- | --- |
| Control vs acute burn | 0.05 |
| Control vs healing | 0.91 |
| Control vs recovered | 0.82 |
| Acute burn vs healing | 0.10 |
| Acute burn vs recovered | 3.50x10^-3^ |
| Healing vs recovering | 0.82 |

**Table S76 – Group comparisons of LDL subfraction 5 cholesterol (L5CH).**

| **Group comparisons** | **Adjusted *p-*value** |
| --- | --- |
| Control vs acute burn | 0.13 |
| Control vs healing | 1.00 |
| Control vs recovered | 1.00 |
| Acute burn vs healing | 1.00 |
| Acute burn vs recovered | 1.00 |
| Healing vs recovering | 1.00 |

**Table S77 – Group comparisons of LDL subfraction 6 cholesterol (L6CH).**

| **Group comparisons** | **Adjusted *p-*value** |
| --- | --- |
| Control vs acute burn | 8.80x10^-3^ |
| Control vs healing | 5.60x10^-4^ |
| Control vs recovered | 4.90x10^-3^ |
| Acute burn vs healing | 1.00 |
| Acute burn vs recovered | 1.00 |
| Healing vs recovering | 1.00 |

**Figure S17 – Box plots of the free cholesterol concentration in the LDL subfractions for the non-burn controls (blue), acute burn injury (red), healing (green) and recovered (green) burns patients.** (A) LDL subfraction 1 free cholesterol (L1FC), (B) LDL subfraction 2 free cholesterol (L2FC), (C) LDL subfraction 3 free cholesterol (L3FC), (D) LDL subfraction 4 free cholesterol (L4FC), (E) LDL subfraction 5 free cholesterol (L5FC), (F) LDL subfraction 6 free cholesterol (L6FC). (**p*<0.05; ***p*<0.01; ****p*<0.001; *****p*<0.0001) determined using Wilcoxon test.

**Table S78 – Group comparisons of LDL subfraction 1 free cholesterol (L1FC).**

| **Group comparisons** | **Adjusted *p-*value** |
| --- | --- |
| Control vs acute burn | 4.30x10^-4^ |
| Control vs healing | 0.05 |
| Control vs recovered | 0.15 |
| Acute burn vs healing | 0.55 |
| Acute burn vs recovered | 0.15 |
| Healing vs recovering | 0.55 |

**Table S79 – Group comparisons of LDL subfraction 2 free cholesterol (L2FC).**

| **Group comparisons** | **Adjusted *p-*value** |
| --- | --- |
| Control vs acute burn | 6.30x10^-10^ |
| Control vs healing | 5.40x10^-3^ |
| Control vs recovered | 1.70x10^-2^ |
| Acute burn vs healing | 4.00x10^-4^ |
| Acute burn vs recovered | 6.80x10^-6^ |
| Healing vs recovering | 0.38 |

**Table S80 – Group comparisons of LDL subfraction 3 free cholesterol (L3FC).**

| **Group comparisons** | **Adjusted *p-*value** |
| --- | --- |
| Control vs acute burn | 6.00x10^-3^ |
| Control vs healing | 0.81 |
| Control vs recovered | 0.43 |
| Acute burn vs healing | 0.02 |
| Acute burn vs recovered | 3.20x10^-6^ |
| Healing vs recovering | 0.24 |

**Table S81 – Group comparisons of LDL subfraction 4 free cholesterol (L4FC).**

| **Group comparisons** | **Adjusted *p-*value** |
| --- | --- |
| Control vs acute burn | 0.15 |
| Control vs healing | 0.37 |
| Control vs recovered | 0.04 |
| Acute burn vs healing | 0.04 |
| Acute burn vs recovered | 2.20x10^-4^ |
| Healing vs recovering | 0.35 |

**Table S82 – Group comparisons of LDL subfraction 5 free cholesterol (L5FC).**

| **Group comparisons** | **Adjusted *p-*value** |
| --- | --- |
| Control vs acute burn | 1.00 |
| Control vs healing | 1.00 |
| Control vs recovered | 1.00 |
| Acute burn vs healing | 1.00 |
| Acute burn vs recovered | 0.91 |
| Healing vs recovering | 1.00 |

**Table S83 – Group comparisons of LDL subfraction 6 free cholesterol (L6FC).**

| **Group comparisons** | **Adjusted *p-*value** |
| --- | --- |
| Control vs acute burn | 0.21 |
| Control vs healing | 9.40x10^-4^ |
| Control vs recovered | 1.40x10^-3^ |
| Acute burn vs healing | 0.15 |
| Acute burn vs recovered | 0.18 |
| Healing vs recovering | 0.82 |

**Figure S18 – Box plots of the phospholipid concentration in the LDL subfractions for the non-burn controls (blue), acute burn injury (red), healing (green) and recovered (green) burns patients.** (A) LDL subfraction 1 phospholipid (L1PL), (B) LDL subfraction 2 phospholipid (L2PL), (C) LDL subfraction 3 phospholipid (L3PL), (D) LDL subfraction 4 phospholipid (L4PL), (E) LDL subfraction 5 phospholipid (L5PL), (F) LDL subfraction 6 phospholipid (L6PL). (**p*<0.05; ***p*<0.01; ****p*<0.001; *****p*<0.0001) determined using Wilcoxon test.

**Table S84 – Group comparisons of LDL subfraction 1 phospholipid (L1PL).**

| **Group comparisons** | **Adjusted *p-*value** |
| --- | --- |
| Control vs acute burn | 9.90x10^-3^ |
| Control vs healing | 0.03 |
| Control vs recovered | 0.05 |
| Acute burn vs healing | 1.00 |
| Acute burn vs recovered | 1.00 |
| Healing vs recovering | 1.00 |

**Table S85 – Group comparisons of LDL subfraction 2 phospholipid (L2PL).**

| **Group comparisons** | **Adjusted *p-*value** |
| --- | --- |
| Control vs acute burn | 6.10x10^-10^ |
| Control vs healing | 3.00x10^-3^ |
| Control vs recovered | 0.02 |
| Acute burn vs healing | 2.50x10^-4^ |
| Acute burn vs recovered | 1.50x10^-6^ |
| Healing vs recovering | 0.26 |

**Table S86 – Group comparisons of LDL subfraction 3 phospholipid (L3PL).**

| **Group comparisons** | **Adjusted *p-*value** |
| --- | --- |
| Control vs acute burn | 5.00x10^-5^ |
| Control vs healing | 0.45 |
| Control vs recovered | 0.57 |
| Acute burn vs healing | 0.01 |
| Acute burn vs recovered | 1.80x10^-6^ |
| Healing vs recovering | 0.21 |

**Table S87 – Group comparisons of LDL subfraction 4 phospholipid (L4PL).**

| **Group comparisons** | **Adjusted *p-*value** |
| --- | --- |
| Control vs acute burn | 0.42 |
| Control vs healing | 0.74 |
| Control vs recovered | 0.42 |
| Acute burn vs healing | 0.40 |
| Acute burn vs recovered | 0.02 |
| Healing vs recovering | 0.51 |

**Table S88 – Group comparisons of LDL subfraction 5 phospholipid (L5PL).**

| **Group comparisons** | **Adjusted *p-*value** |
| --- | --- |
| Control vs acute burn | 0.23 |
| Control vs healing | 0.88 |
| Control vs recovered | 0.88 |
| Acute burn vs healing | 1.00 |
| Acute burn vs recovered | 1.00 |
| Healing vs recovering | 1.00 |

**Table S89 – Group comparisons of LDL subfraction 6 phospholipid (L6PL).**

| **Group comparisons** | **Adjusted *p-*value** |
| --- | --- |
| Control vs acute burn | 0.03 |
| Control vs healing | 4.60x10^-3^ |
| Control vs recovered | 0.02 |
| Acute burn vs healing | 1.00 |
| Acute burn vs recovered | 1.00 |
| Healing vs recovering | 1.00 |

**Figure S19 – Box plots of the Apolipoprotein B100 concentration in the LDL subfractions for the non-burn controls (blue), acute burn injury (red), healing (green) and recovered (green) burns patients.** (A) LDL subfraction 1 Apo-B (L1AB), (B) LDL subfraction 2 Apo-B (L2AB), (C) LDL subfraction 3 Apo-B (L3AB), (D) LDL subfraction 4 Apo-B (L4AB), (E) LDL subfraction 5 Apo-B (L5AB), (F) LDL subfraction 6 Apo-B (L6AB). (**p*<0.05; ***p*<0.01; ****p*<0.001; *****p*<0.0001) determined using Wilcoxon test.

**Table S90 – Group comparisons of LDL subfraction 1 Apo-B (L1AB).**

| **Group comparisons** | **Adjusted *p-*value** |
| --- | --- |
| Control vs acute burn | 0.77 |
| Control vs healing | 0.23 |
| Control vs recovered | 0.41 |
| Acute burn vs healing | 0.77 |
| Acute burn vs recovered | 0.77 |
| Healing vs recovering | 0.77 |

**Table S91 – Group comparisons of LDL subfraction 2 Apo-B (L2AB).**

| **Group comparisons** | **Adjusted *p-*value** |
| --- | --- |
| Control vs acute burn | 5.60x10^-9^ |
| Control vs healing | 2.70x10^-3^ |
| Control vs recovered | 0.01 |
| Acute burn vs healing | 1.50x10^-3^ |
| Acute burn vs recovered | 4.50x10^-5^ |
| Healing vs recovering | 0.23 |

**Table S92 – Group comparisons of LDL subfraction 3 Apo-B (L3AB).**

| **Group comparisons** | **Adjusted *p-*value** |
| --- | --- |
| Control vs acute burn | 8.80x10^-4^ |
| Control vs healing | 0.36 |
| Control vs recovered | 0.51 |
| Acute burn vs healing | 0.12 |
| Acute burn vs recovered | 4.10x10^-5^ |
| Healing vs recovering | 0.12 |

**Table S93 – Group comparisons of LDL subfraction 4 Apo-B (L4AB).**

| **Group comparisons** | **Adjusted *p-*value** |
| --- | --- |
| Control vs acute burn | 0.85 |
| Control vs healing | 0.89 |
| Control vs recovered | 0.86 |
| Acute burn vs healing | 0.86 |
| Acute burn vs recovered | 0.09 |
| Healing vs recovering | 0.86 |

**Table S94 – Group comparisons of LDL subfraction 5 Apo-B (L5AB).**

| **Group comparisons** | **Adjusted *p-*value** |
| --- | --- |
| Control vs acute burn | 1.00 |
| Control vs healing | 1.00 |
| Control vs recovered | 1.00 |
| Acute burn vs healing | 1.00 |
| Acute burn vs recovered | 1.00 |
| Healing vs recovering | 1.00 |

**Table S95 – Group comparisons of LDL subfraction 6 Apo-B (L6AB).**

| **Group comparisons** | **Adjusted *p-*value** |
| --- | --- |
| Control vs acute burn | 1.30x10^-4^ |
| Control vs healing | 1.10x10^-4^ |
| Control vs recovered | 5.90x10^-4^ |
| Acute burn vs healing | 1.00 |
| Acute burn vs recovered | 1.00 |
| Healing vs recovering | 1.00 |

**Figure S20 – Box plots of the triglyceride concentration in the HDL subfractions for the non-burn controls (blue), acute burn injury (red), healing (green) and recovered (green) burns patients.** (A) HDL subfraction 1 triglyceride (H1TG), (B) HDL subfraction 2 triglyceride (H2TG), (C) HDL subfraction 3 triglyceride (H3TG), (D) HDL subfraction 4 triglyceride (H4TG). (**p*<0.05; ***p*<0.01; ****p*<0.001; *****p*<0.0001) determined using Wilcoxon test.

**Table S96 – Group comparisons of HDL subfraction 1 triglyceride (H1TG).**

| **Group comparisons** | **Adjusted *p-*value** |
| --- | --- |
| Control vs acute burn | 1.00 |
| Control vs healing | 0.45 |
| Control vs recovered | 1.00 |
| Acute burn vs healing | 0.73 |
| Acute burn vs recovered | 1.00 |
| Healing vs recovering | 1.00 |

**Table S97 – Group comparisons of HDL subfraction 2 triglyceride (H2TG).**

| **Group comparisons** | **Adjusted *p-*value** |
| --- | --- |
| Control vs acute burn | 0.93 |
| Control vs healing | 0.04 |
| Control vs recovered | 0.26 |
| Acute burn vs healing | 0.03 |
| Acute burn vs recovered | 0.26 |
| Healing vs recovering | 0.54 |

**Table S98 – Group comparisons of HDL subfraction 3 triglyceride (H3TG).**

| **Group comparisons** | **Adjusted *p-*value** |
| --- | --- |
| Control vs acute burn | 1.00 |
| Control vs healing | 0.09 |
| Control vs recovered | 0.20 |
| Acute burn vs healing | 0.02 |
| Acute burn vs recovered | 0.09 |
| Healing vs recovering | 1.00 |

**Table S99 – Group comparisons of HDL subfraction 4 triglyceride (H4TG).**

| **Group comparisons** | **Adjusted *p-*value** |
| --- | --- |
| Control vs acute burn | 0.27 |
| Control vs healing | 0.62 |
| Control vs recovered | 0.18 |
| Acute burn vs healing | 0.18 |
| Acute burn vs recovered | 7.30x10^-4^ |
| Healing vs recovering | 0.32 |

**Figure S21 – Box plots of the cholesterol concentration in the HDL subfractions for the non-burn controls (blue), acute burn injury (red), healing (green) and recovered (green) burns patients.** (A) HDL subfraction 1 cholesterol (H1CH), (B) HDL subfraction 2 cholesterol (H2CH), (C) HDL subfraction 3 cholesterol (H3CH), (D) HDL subfraction 4 cholesterol (H4CH). (**p*<0.05; ***p*<0.01; ****p*<0.001; *****p*<0.0001) determined using Wilcoxon test.

**Table S100 – Group comparisons of HDL subfraction 1 cholesterol (H1CH).**

| **Group comparisons** | **Adjusted *p-*value** |
| --- | --- |
| Control vs acute burn | 1.70x10^-6^ |
| Control vs healing | 0.03 |
| Control vs recovered | 0.22 |
| Acute burn vs healing | 0.20 |
| Acute burn vs recovered | 4.00x10^-3^ |
| Healing vs recovering | 0.24 |

**Table S101 – Group comparisons of HDL subfraction 2 cholesterol (H2CH).**

| **Group comparisons** | **Adjusted *p-*value** |
| --- | --- |
| Control vs acute burn | 2.90x10^-11^ |
| Control vs healing | 4.70x10^-5^ |
| Control vs recovered | 4.20x10^-3^ |
| Acute burn vs healing | 0.07 |
| Acute burn vs recovered | 8.20x10^-6^ |
| Healing vs recovering | 0.05 |

**Table S102 – Group comparisons of HDL subfraction 3 cholesterol (H3CH).**

| **Group comparisons** | **Adjusted *p-*value** |
| --- | --- |
| Control vs acute burn | 1.30x10^-8^ |
| Control vs healing | 0.03 |
| Control vs recovered | 0.64 |
| Acute burn vs healing | 0.03 |
| Acute burn vs recovered | 2.10x10^-6^ |
| Healing vs recovering | 0.07 |

**Table S103 – Group comparisons of HDL subfraction 4 cholesterol (H4CH).**

| **Group comparisons** | **Adjusted *p-*value** |
| --- | --- |
| Control vs acute burn | 0.23 |
| Control vs healing | 0.02 |
| Control vs recovered | 0.23 |
| Acute burn vs healing | 5.80x10^-5^ |
| Acute burn vs recovered | 3.00x10^-3^ |
| Healing vs recovering | 0.25 |

**Figure S22 – Box plots of the free cholesterol concentration in the HDL subfractions for the non-burn controls (blue), acute burn injury (red), healing (green) and recovered (green) burns patients.** (A) HDL subfraction 1 free cholesterol (H1FC), (B) HDL subfraction 2 free cholesterol (H2FC), (C) HDL subfraction 3 free cholesterol (H3FC), (D) HDL subfraction 4 free cholesterol (H4FC). (**p*<0.05; ***p*<0.01; ****p*<0.001; *****p*<0.0001) determined using Wilcoxon test.

**Table S104 – Group comparisons of HDL subfraction 1 free cholesterol (H1FC).**

| **Group comparisons** | **Adjusted *p-*value** |
| --- | --- |
| Control vs acute burn | 4.10x10^-11^ |
| Control vs healing | 4.60x10^-3^ |
| Control vs recovered | 0.14 |
| Acute burn vs healing | 0.02 |
| Acute burn vs recovered | 2.80x10^-6^ |
| Healing vs recovering | 0.14 |

**Table S105 – Group comparisons of HDL subfraction 2 free cholesterol (H2FC).**

| **Group comparisons** | **Adjusted *p-*value** |
| --- | --- |
| Control vs acute burn | 6.80x10^-12^ |
| Control vs healing | 7.70x10^-4^ |
| Control vs recovered | 0.32 |
| Acute burn vs healing | 0.07 |
| Acute burn vs recovered | 1.40x10^-8^ |
| Healing vs recovering | 0.02 |

**Table S106 – Group comparisons of HDL subfraction 3 free cholesterol (H3FC).**

| **Group comparisons** | **Adjusted *p-*value** |
| --- | --- |
| Control vs acute burn | 6.90x10^-6^ |
| Control vs healing | 0.60 |
| Control vs recovered | 0.60 |
| Acute burn vs healing | 3.60x10^-3^ |
| Acute burn vs recovered | 1.40x10^-6^ |
| Healing vs recovering | 0.32 |

**Table S107 – Group comparisons of HDL subfraction 4 free cholesterol (H4FC).**

| **Group comparisons** | **Adjusted *p-*value** |
| --- | --- |
| Control vs acute burn | 0.53 |
| Control vs healing | 5.70x10^-5^ |
| Control vs recovered | 1.30x10^-4^ |
| Acute burn vs healing | 1.80x10^-3^ |
| Acute burn vs recovered | 1.80x10^-3^ |
| Healing vs recovering | 0.94 |

**Figure S23 – Box plots of the phospholipid concentration in the HDL subfractions for the non-burn controls (blue), acute burn injury (red), healing (green) and recovered (green) burns patients.** (A) HDL subfraction 1 phospholipid (H1PL), (B) HDL subfraction 2 phospholipid (H2PL), (C) HDL subfraction 3 phospholipid (H3PL), (D) HDL subfraction 4 phospholipid (H4PL). (**p*<0.05; ***p*<0.01; ****p*<0.001; *****p*<0.0001) determined using Wilcoxon test.

**Table S108 – Group comparisons of HDL subfraction 1 phospholipid (H1PL).**

| **Group comparisons** | **Adjusted *p-*value** |
| --- | --- |
| Control vs acute burn | 2.00x10^-7^ |
| Control vs healing | 3.90x10^-3^ |
| Control vs recovered | 0.14 |
| Acute burn vs healing | 0.27 |
| Acute burn vs recovered | 4.00x10^-3^ |
| Healing vs recovering | 0.25 |

**Table S109 – Group comparisons of HDL subfraction 2 phospholipid (H2PL).**

| **Group comparisons** | **Adjusted *p-*value** |
| --- | --- |
| Control vs acute burn | 4.50x10^-10^ |
| Control vs healing | 8.10x10^-7^ |
| Control vs recovered | 8.00x10^-3^ |
| Acute burn vs healing | 0.92 |
| Acute burn vs recovered | 1.60x10^-3^ |
| Healing vs recovering | 0.01 |

**Table S110 – Group comparisons of HDL subfraction 3 phospholipid (H3PL).**

| **Group comparisons** | **Adjusted *p-*value** |
| --- | --- |
| Control vs acute burn | 3.80x10^-7^ |
| Control vs healing | 2.60x10^-3^ |
| Control vs recovered | 0.31 |
| Acute burn vs healing | 0.20 |
| Acute burn vs recovered | 1.00x10^-3^ |
| Healing vs recovering | 0.11 |

**Table S111 – Group comparisons of HDL subfraction 4 phospholipid (H4PL).**

| **Group comparisons** | **Adjusted *p-*value** |
| --- | --- |
| Control vs acute burn | 0.36 |
| Control vs healing | 0.18 |
| Control vs recovered | 0.36 |
| Acute burn vs healing | 6.90x10^-3^ |
| Acute burn vs recovered | 0.02 |
| Healing vs recovering | 0.74 |

**Figure S24 – Box plots of the Apolipoprotein-A1 concentration in the HDL subfractions for the non-burn controls (blue), acute burn injury (red), healing (green) and recovered (green) burns patients.** (A) HDL subfraction 1 Apo-A1 (H1A1), (B) HDL subfraction 2 Apo-A1 (H2A1), (C) HDL subfraction 3 Apo-A1 (H3A1), (D) HDL subfraction 4 Apo-A1 (H4A1). (**p*<0.05; ***p*<0.01; ****p*<0.001; *****p*<0.0001) determined using Wilcoxon test.

**Table S112 – Group comparisons of HDL subfraction 1 Apo-A1 (H1A1).**

| **Group comparisons** | **Adjusted *p-*value** |
| --- | --- |
| Control vs acute burn | 9.90x10^-12^ |
| Control vs healing | 1.40x10^-4^ |
| Control vs recovered | 2.90x10^-3^ |
| Acute burn vs healing | 0.06 |
| Acute burn vs recovered | 1.10x10^-3^ |
| Healing vs recovering | 0.24 |

**Table S113 – Group comparisons of HDL subfraction 2 Apo-A1 (H2A1).**

| **Group comparisons** | **Adjusted *p-*value** |
| --- | --- |
| Control vs acute burn | 5.20x10^-6^ |
| Control vs healing | 1.00x10^-3^ |
| Control vs recovered | 0.45 |
| Acute burn vs healing | 0.53 |
| Acute burn vs recovered | 2.20x10^-3^ |
| Healing vs recovering | 0.03 |

**Table S114 – Group comparisons of HDL subfraction 3 Apo-A1 (H3A1).**

| **Group comparisons** | **Adjusted *p-*value** |
| --- | --- |
| Control vs acute burn | 2.90x10^-4^ |
| Control vs healing | 0.06 |
| Control vs recovered | 0.80 |
| Acute burn vs healing | 0.42 |
| Acute burn vs recovered | 5.90x10^-3^ |
| Healing vs recovering | 0.14 |

**Table S115 – Group comparisons of HDL subfraction 4 Apo-A1 (H4A1).**

| **Group comparisons** | **Adjusted *p-*value** |
| --- | --- |
| Control vs acute burn | 0.39 |
| Control vs healing | 0.01 |
| Control vs recovered | 0.16 |
| Acute burn vs healing | 0.42 |
| Acute burn vs recovered | 2.40x10^-5^ |
| Healing vs recovering | 0.39 |

**Figure S25 – Box plots of the Apolipoprotein-A2 concentration in the HDL subfractions for the non-burn controls (blue), acute burn injury (red), healing (green) and recovered (green) burns patients.** (A) HDL subfraction 1 Apo-A2 (H1A2), (B) HDL subfraction 2 Apo-A2 (H2A2), (C) HDL subfraction 3 Apo-A2 (H3A2), (D) HDL subfraction 4 Apo-A2 (H4A2). (**p*<0.05; ***p*<0.01; ****p*<0.001; *****p*<0.0001) determined using Wilcoxon test.

**Table S116 – Group comparisons of HDL subfraction 1 Apo-A2 (H1A2).**

| **Group comparisons** | **Adjusted *p-*value** |
| --- | --- |
| Control vs acute burn | 1.30x10^-9^ |
| Control vs healing | 1.30x10^-5^ |
| Control vs recovered | 0.01 |
| Acute burn vs healing | 0.79 |
| Acute burn vs recovered | 1.20x10^-3^ |
| Healing vs recovering | 0.03 |

**Table S117 – Group comparisons of HDL subfraction 2 Apo-A2 (H2A2).**

| **Group comparisons** | **Adjusted *p-*value** |
| --- | --- |
| Control vs acute burn | 4.80x10^-14^ |
| Control vs healing | 1.10x10^-8^ |
| Control vs recovered | 3.30x10^-4^ |
| Acute burn vs healing | 0.41 |
| Acute burn vs recovered | 2.20x10^-4^ |
| Healing vs recovering | 0.01 |

**Table S118 – Group comparisons of HDL subfraction 3 Apo-A2 (H3A2).**

| **Group comparisons** | **Adjusted *p-*value** |
| --- | --- |
| Control vs acute burn | 3.80x10^-15^ |
| Control vs healing | 6.70x10^-8^ |
| Control vs recovered | 1.30x10^-3^ |
| Acute burn vs healing | 0.10 |
| Acute burn vs recovered | 1.30x10^-3^ |
| Healing vs recovering | 0.10 |

**Table S119 – Group comparisons of HDL subfraction 4 Apo-A2 (H4A2).**

| **Group comparisons** | **Adjusted *p-*value** |
| --- | --- |
| Control vs acute burn | 0.13 |
| Control vs healing | 0.03 |
| Control vs recovered | 0.10 |
| Acute burn vs healing | 1.30x10^-4^ |
| Acute burn vs recovered | 1.90x10^-3^ |
| Healing vs recovering | 0.57 |

**Figure S26 – Validation of the OPLS-DA model by permutation testing (Figure 2).** The OPLS-DA model was evaluated using 1000 permutation tests to assess model robustness and the potential for overfitting. (A) Model summary showing the cumulative R²Y and Q² values for the predictive (p1) and orthogonal (o1–o5) components. (B) Permutation test comparing the original model (rightmost point) with models generated after random permutation of the class labels. The empirical permutation *p*-values were pR²Y = 0.001 and pQ² = 0.001, indicating that the observed model performance was significantly better than expected by chance. (C) Observation diagnostics showing score distance (SD) versus orthogonal distance (OD) for individual samples; dashed lines indicate the model thresholds used to identify potential outliers. (D) OPLS-DA scores plot showing separation between the Acute and recovered groups. The model explained 82.0% of the variation in X (R²X = 0.82), 62.7% of the variation in Y (R²Y = 0.627), and had a predictive ability of Q² = 0.506. (E) Receiver operating characteristic (ROC) and precision–recall (PR) curve analysis of the classification model. The model achieved high diagnostic performance with an AUC-ROC of 0.97 and an AUC-PR of 0.98, indicating excellent ability to distinguish Recovered samples from Control samples with high sensitivity and precision.

**Figure S27 – LDL particle numbers for the control and recovered patients.** Percentages are determined by rescaling all to 100% from table S120.

**Table S120 – Percentage composition of the LDL by subfraction size.**

| **Lipoprotein subfraction** | **Percentage median of LDL subfraction for the control group ± standard deviation** | **Percentage median of LDL subfraction for the recovered group ± standard deviation** | ***p-*value, determined using the median values of each group** |
| --- | --- | --- | --- |
| LDL1 subfraction (L1PN) | 21.37 ± 5.15 | 18.74 ± 4.85 | 2.78x10^-2^ |
| LDL2 subfraction (L2PN) | 26.33 ± 9.58 | 20.26 ± 4.12 | 1.50x10^-7^ |
| LDL3 subfraction (L3PN) | 19.26 ± 3.52 | 19.66 ± 3.70 | 9.78x10^-1^ |
| LDL4 subfraction (L4PN) | 10.67 ± 6.28 | 12.90 ± 4.80 | 1.88x10^-3^ |
| LDL5 subfraction (L5PN) | 8.74 ±5.21 | 9.87 ± 4.65 | 3.49x10^-1^ |
| LDL6 subfraction (L6PN) | 12.34 ± 8.99 | 17.63 ± 7.00 | 3.01x10^-4^ |

**Table S121 – Percentage composition of lipids within the main lipoprotein fractions, VLDL, IDL, LDL and HDL.** CE – cholesterol ester, FC – free cholesterol, PL – phospholipid, TG – triglyceride.

| **Lipoprotein fraction lipid content** | **Percentage of lipoprotein fraction for the control group ± standard deviation** | **Percentage of lipoprotein fraction for the recovered group ± standard deviation** | ***p-*value** |
| --- | --- | --- | --- |
| VLDL CE | 8.13 ± 3.74 | 6.03 ± 3.74 | 9.38x10^-4^ |
| VLDL FC | 7.45 ± 1.35 | 7.86 ± 1.30 | 0.40 |
| VLDL PL | 19.64 ± 4.14 | 18.49 ± 2.45 | 1.21x10^-4^ |
| VLDL TG | 64.52 ± 5.82 | 67.50 ± 5.02 | 9.79x10^-6^ |
|  |  |  |  |
| IDL CE | 29.83 ± 13.78 | 39.23 ±15.18 | 2.41x10^-3^ |
| IDL FC | 12.23 ± 4.67 | 14.57 ± 5.08 | 9.16x10^-2^ |
| IDL PL | 28.07 ± 16.34 | 22.51 ± 15.14 | 3.23x10^-2^ |
| IDL TG | 25.04 ± 19.78 | 20.38 ± 14.76 | 0.10 |
|  |  |  |  |
| LDL CE | 41.18 ± 4.31 | 38.24 ± 3.30 | 5.51x10^-4^ |
| LDL FC | 18.21 ± 1.67 | 18.60 ± 1.35 | 4.60x10^-2^ |
| LDL PL | 33.38 ± 0.93 | 33.68 ± 0.92 | 0.57 |
| LD TG | 7.08 ± 3.32 | 8.98 ± 3.13 | 2.28x10^-3^ |
|  |  |  |  |
| HDL CE | 30.7 ± 3.30 | 28.98 ± 2.47 | 2.69x10^-4^ |
| HDL FC | 8.98 ± 1.29 | 9.71 ± 1.01 | 5.84x10^-3^ |
| HDL PL | 53.92 ±1.63 | 55.03 ± 1.83 | 6.30x10^-3^ |
| HDL TG | 5.91 ± 2.62 | 6.26 ± 2.60 | 0.27 |
